# Enteric methane emission of dairy cows supplemented with iodoform in a dose–response study

**DOI:** 10.1038/s41598-023-38149-y

**Published:** 2023-08-07

**Authors:** Mirka Thorsteinsson, Peter Lund, Martin Riis Weisbjerg, Samantha Joan Noel, Anna Amanda Schönherz, Anne Louise Frydendahl Hellwing, Hanne Helene Hansen, Mette Olaf Nielsen

**Affiliations:** 1 Department of Animal and Veterinary Sciences, AU Viborg – Research Centre Foulum, Aarhus University, 8830 Tjele, Denmark; 2 iCLIMATE - Interdisciplinary Centre for Climate Change, Aarhus University, 8830 Tjele, Denmark; 3 CBIO - Centre for Circular Bioeconomy, Aarhus University, 8830 Tjele, Denmark; 4 Department of Veterinary and Animal Sciences, University of Copenhagen, 1870 Frederiksberg, Denmark

**Keywords:** Microbiology, Physiology, Environmental sciences, Biomarkers, Gastroenterology

## Abstract

Enteric methane (CH_4_) emission is one of the major greenhouse gasses originating from cattle. Iodoform has in studies been found to be a potent mitigator of rumen CH_4_ formation in vitro. This study aimed to quantify potential of iodoform as an anti-methanogenic feed additive for dairy cows and investigate effects on feed intake, milk production, feed digestibility, rumen microbiome, and animal health indicators. The experiment was conducted as a 4 × 4 Latin square design using four lactating rumen, duodenal, and ileal cannulated Danish Holstein dairy cows. The treatments consisted of four different doses of iodoform (1) 0 mg/day, (2) 320 mg/day, (3) 640 mg/day, and (4) 800 mg/day. Iodoform was supplemented intra-ruminally twice daily. Each period consisted of 7-days of adaptation, 3-days of digesta and blood sampling, and 4-days of gas exchange measurements using respiration chambers. Milk yield and dry matter intake (DMI) were recorded daily. Rumen samples were collected for microbial analyses and investigated for fermentation parameters. Blood was sampled and analyzed for metabolic and health status indicators. Dry matter intake and milk production decreased linearly by maximum of 48% and 33%, respectively, with increasing dose. Methane yield (g CH_4_/kg DMI) decreased by maximum of 66%, while up to 125-fold increases were observed in hydrogen yield (g H_2_/kg DMI) with increasing dose of iodoform. Total tract digestibility of DM, OM, CP, C, NDF, and starch were unaffected by treatments, but large shifts, except for NDF, were observed for ruminal to small intestinal digestion of the nutrients. Some indicators of disturbed rumen microbial activity and fermentation dynamics were observed with increasing dose, but total number of ruminal bacteria was unaffected by treatment. Serum and plasma biomarkers did not indicate negative effects of iodoform on cow health. In conclusion, iodoform was a potent mitigator of CH_4_ emission. However, DMI and milk production were negatively affected and associated with indications of depressed ruminal fermentation. Future studies might reveal if depression of milk yield and feed intake can be avoided if iodoform is continuously administered by mixing it into a total mixed ration.

## Introduction

Methane (CH4) is one of the major greenhouse gases originating from cattle. The global warming potential in a 100 years perspective is 28 times higher compared to carbon dioxide (CO2). Hence, the contribution from cattle to greenhouse gas emissions and climate change is substantial^1,2^. Methane from ruminant animals originates primarily from microbial fermentation of feeds in the rumen. During this fermentation, microbes produce CO_2_ and hydrogen (H_2_), which are converted to CH_4_ by archaea, a special domain of microorganisms. Methanogenesis efficiently lowers the partial pressure in the rumen of hydrogen (H_2_), which serves as an electron donor in the process, where CO_2_ and H_2_ are converted into CH_4_ and H_2_O by the methanogenic archaea^3^. This continuous removal sustains ruminal fermentation by eliminating the inhibitory effect of H_2_ on the microbiota^4^. Thus, an increased ruminal H_2_ pressure may affect fiber digestion, dry matter intake (DMI), and animal productivity negatively^5^. Under substantial methanogenesis inhibition, H_2_ can also be eructated in significant amounts^6,7^.

Historically, halogenated compounds such as trichloroethyl adipate, trichloroethyl pivalate, bromoform, and chloroform have been shown to be potent inhibitors of CH_4_ formation in both in vitro and in vivo studies^8–10^. The anti-methanogenic activity is usually related to the number of halogens on the molecule with iodine-containing compounds being the most efficient followed by brominated and chlorinated analogs^11^. The suggested mechanism involves the irreversible reaction of the halogenated compounds with reduced vitamin B_12_ to inhibit the cobamide-dependent methyl group transfer and by blocking function of corrinoid enzymes in the methanogenesis process^12–14^. Another possible mechanism is the competitive inhibition of CH_4_ production by serving as competing terminal electron acceptors^14,15^.

Mitsumori et al.^16^ found that bromochloromethane (CH_2_BrCl) resulted in an inhibition of methanogenesis of around 80% in goats, which was also associated with a dramatic increase in H_2_ emission. The supplementation had no effects on DMI or nutrient digestibility^16^. Similar inhibition of the methanogenesis by bromochloromethane has been observed in other studies^17,18^. However, due to its ozone-depleting capacity, bromochloromethane is banned from commercial use in many countries^14,19^. Another halomethane, chloroform (CHCl_3_), is also able to inhibit methanogenesis to a similar extent as bromochloromethane^20,21^. Chloroform is normally not considered to be ozone-depleting due to its short lifetime and predominantly natural origin^22^, but it is recognized as a carcinogenic substance^19,23^. In constrast, iodoform (CHI_3_) is a halomethane that is considered to be neither ozone-depleting nor carcinogenic, and it is approved for use in the pharmaceutical industry with no upper tolerance level, but it is as yet not approved as a feed additive^19^. It has previously been observed that iodoform is a potent inhibitor of CH_4_ production in vitro^8^. However, no studies have tested iodoform when fed to dairy cows.

The aims of this experiment were to quantifying the anti-methanogenic potential of iodoform when pulse-dosed intra-ruminally to dairy cows in a dose–response study and to study associated effects on feed intake, milk production, nutrient digestibility, microbiome, and animal health indicators. It was hypothesized that increasing doses of iodoform would decrease CH_4_ linearly, while at the same time increase H_2_ emission, without affecting feed intake or milk production from dairy cows.

## Materials and methods

### Experimental design

The experiment was conducted at Aarhus University, AU Viborg - Research Centre Foulum, Denmark, and complied with the guidelines set out by the Danish Ministry of Environment and Food with respect to animal experimentation and care of animals under study (act 474 of 15th of May 2014 and executive order 2028 of 14th of December 2020) and under consideration of the ARRIVE Guidelines. The experimental protocol was approved by the Danish Animal Experiments Inspectorate (license no. 2018-15-0201-01495). Four lactating Danish Holstein dairy cows were assigned to 1 of 4 levels (0, 320, 640, or 800 mg/day) of iodoform according to a 4 × 4 Latin square design. Each of the four experimental periods consisted of 7 days of adaptation, 3 days of digesta and blood sampling, and 4 days of gas exchange measurement. To account for the relatively short adaptation period, the cows were rumen inoculated on day 1–2 in every period with rumen content from two non-experimental cannulated cows that were fed the same ration as the experimental cows. Originally, the highest dose of iodoform was intended to be 1080 mg/day. However, this treatment was cessationed after 11 days in period 1 due an unacceptable decrease of feed intake. Thus, the highest dosage was reduced to 800 mg/day in the remaining periods, and the cow initially assigned to the highest dose was replaced by a different cow.

### Animals and housing

Four multiparous cows (two 2nd parity and two 3rd parity), which had previously been fitted with rumen, duodenal, and ileal cannulas for the collection of digesta, were used. At the beginning of the experiment, average ± SD milk yield was 33.1 ± 2.10 kg/day, days in milk was 210 ± 66 days, and BW was 666 ± 60 kg. The cows were housed in individual pens (400 × 450 cm) with slatted floor and a cubicle bedded with mattress and sawdust. The cows were milked twice daily at 06.30 and 16.30 h.

### Diet and feeding

A partial mixed ration (PMR) was prepared once a day in the morning and fed to the cows on an ad libitum basis in two daily meals given at 7.15 and 17.15 h with 3–5 kg feed residues per cow per day with approximately 40% of the daily ration provided in the morning and 60% in the afternoon. Feed residues were weighed daily before morning feeding. The ration was formulated according to the NorFor feed evaluation system^24^ for Danish Holstein dairy cows producing 11,700 kg ECM per year. Maize silage and both spring growth and first regrowth grass/clover silage (perennial ryegrass, hybrid ryegrass, red clover, and white clover) from the same silo bunks throughout the experiment were included as roughages and soybean meal, sugar beet pulp, rapeseed cake, barley, and mineral supplements were included as concentrates (Table 1). Half of the daily dose of iodoform, dissolved in 20 mL 99% ethanol and mixed with 500 g ± 1 g of concentrate (KomKalv, DLG a.m.b.a., Denmark), was administrated intra-ruminally by manually mixing it into the rumen content during milking at 06:30 and 16:30 h. As a fixed amount of concentrate was used to ensure proper mixing of iodoform into the concentrate before dosing, the roughage share of the total DMI was dependent on the PMR intake of the individual cow. The nutrient flow in the digestive tract was determined using chromium(III)oxide (Cr_2_O_3_) and titanium dioxide (TiO_2_) as external markers. These were weighed in degradable paper bags (10.0 g of Cr_2_O_3_ and 13.0 g TiO_2_) and dosed directly to the rumen twice daily coinciding with the administration of iodoform. The cows had free access to water, and the amount of ingested water was measured by a water-meter (Brødrene Dahl, Brøndby, Denmark) during the chamber period.

**Table 1:** Dietary composition and chemical composition (in percentage of DM), unless otherwise stated, of the PMR and concentrate fed to the cows given iodoform additive.

Item		
	PMR	Concentrate
Dietary composition, % of DM		
Maize silage	25.3	–
Grass/clover silage, 1. regrowth	14.4	–
Grass/clover silage, spring growth	13.6	–
Sugar beet pulp	14.7	30.2
Spring barley	11.7	–
Rapeseed cakes	9.33	6.50
Soybean meal	9.33	6.00
Distillers grain	–	20.0
Wheat bran	–	10.0
Wheat	–	9.30
Rapeseed meal	–	5.40
Grass pellets (artificially dried)	–	3.00
Citrus pulp	–	2.00
Rye	–	2.00
Sunflower meal	–	2.00
Molasses	–	1.65
Mineral and vitamin premix^1^	1.62	1.95
Chemical composition, % of DM		
DM, % of fresh feed	44.0	98.4
Ash	7.54	6.09
CP	16.0	20.3
Crude fat	3.95	3.80
Starch	15.3	13.0
NDF	31.9	28.6
ADF	18.5	14.9
ADL	1.64	3.07
NE_L20_, MJ/kg of DM^2^	6.68	7.73

^1^Vilofoss A/S Komix Type 3, declared macro mineral composition (g/kg DM): Ca = 147, Mg = 141, Na = 116, S = 1. Added vitamins and micro minerals (per kg DM): vitamin A = 600,000.10 IU, vitamin D3 = 190,000.10 IU, vitamin E = 4000 IU, Mn = 4000 mg, Cu = 1500 mg, Zn = 4500 mg, I = 225, Co = 25 mg, Se = 50 mg in combination with Vilofoss Suplex ADE, analyzed/declared macro mineral composition (g/kg DM): Ca = 139, Mg = 91, Na = 95. Added vitamins and micro minerals (per kg DM): vitamin A = 900,000 IU, vitamin D3 = 200,000 IU, vitamin E = 2000 IU, Se = 50 mg.

^2^Standard feed value for net energy for lactation at 20 kg DMI. Calculated according to NorFor^24^.

### Rumen inoculation

On d 1 and 2 in each experimental period, the cows were inoculated at 06.45 h with rumen content from two non-experimental cannulated cows that were fed the same ration as the experimental cows. This was done to minimize any potential carry-over effects on rumen microbiota due to treatments in the previous period. Rumen content (12 kg in total of which 6 kg originated from each non-experimental cow) was introduced to each experimental cow through the rumen cannula and mixed with the upper layer of the rumen content.

### Rumen, digesta, feces, urine, blood, and milk sampling

In each period, rumen fluid, duodenal and ileal content, urine, and feces were collected over 8 sampling times during 3 days (at 9.00 and 18.00 h on day 8; at 03.00, 12.00 and 21.00 h on day 9; and 06.00, 15.00 and 00.00 on day 10) to cover every third hour of a 24 h day. During the sampling period, 8 duodenal (0.5 L) and ileal (0.2 L) samples were collected in plastic bags attached to the cannulas. Fecal (0.35 L) samples were collected during voluntary defecation or by grab sampling from the rectum. Digesta samples were pooled across all sampling times and stored at − 20 °C until analyzed.

Rumen fluid (30 mL) was sampled from the ventral ruminal sac using a 50 mL syringe attached to a 90-cm steel rumen sampler device (Bar Diamond Inc., Parma Idaho, USA). Immediately after sampling, pH was measured in rumen fluid using a digital pH-meter (Meterlab PHM 220, Radiometer, Brønshøj, Denmark). Samples were stored at − 20 °C for later analysis of volatile fatty acids (VFA), l-lactate, glucose, and ammonia (NH_3_) concentration. Simultaneously with the sampling of rumen liquid, redox-potential was measured in the dorsal rumen using a redox meter with a platinum electrode (Intellical MTC101 ORP/redox electrode, Hach, Germany). The electrode was inserted into the rumen content 10 cm below the surface of the rumen mat, and redox potential was recorded after 2 min of stabilizing. Urine was collected at all sampling times during voluntary urinations or upon manual stimulation of the pelvic region. The pH was measured immediately after collection using the same digital pH-meter, and samples were stored at − 20 °C until analyzed for content of creatinine.

Blood was sampled by venipuncture from the tail vein on d 8 at 8.00 and on d 10 at 14.00 h and collected in Na-heparin vacutainers (Greiner Bio-One GmbH, Kremsmünster, Austria) for subsequent determination of thyroxine (T4). The tubes were centrifuged at 3000×*g*_av_ at 4 °C for 20 min. Additionally, blood was drawn into serum vacutainers (Greiner Bio-One GmbH, Kremsmünster, Austria) for determination of urea, glucose, β-OH-butyrate (BHB), non-esterified fatty acids (NEFA), bile acid, total protein, albumin, aspartate aminotransferase (AST), gamma-glutamyl transferase (g-GT), glutamate dehydrogenase (GLDH), and total bilirubin. The samples were left to coagulate for at least 1 h at room temperature before being centrifuged at 1300×*g*_av_ at 20 °C for 10 min. Plasma and serum samples were transferred to cryotubes and stored at − 20 °C until analyzed.

Milk yield was recorded daily throughout the experiment. The composition of the milk was determined on day 12 and 13. Dry matter intake of cows was measured from day 8–14 on a daily basis by weighing the amount of allocated feed and feed residues followed by determination of feed and residue DM contents, while feed intake was recorded throughout the experiment. The nutrient composition of the TMR was determined during the sampling period by pooling the samples from each day across the period.

### Gas exchange measurements

Gas exchange was measured on d 11–14 using four individual transparent polycarbonate respiration chambers based on open-circuit indirect calorimetry, modified from ALF Hellwing, et al.^25^. The chambers were placed in a square in a separate barn to allow visual contact between the cows. Inside dimensions of the chambers measured 415 (length) × 270 (width) × 234 cm (height), resulting in a volume of 28.4 m^3^. Airflow was measured using a mass flow meter (HFM-200 with laminar flow element, Teledyne Hastings Instruments, Hampton, Virginia, USA). The concentrations of gases (CH_4_, CO_2_, O_2_, and H_2_; Columbus Instruments, Columbus, Ohio, USA) in outlet air, and temperature, humidity, and pressure (Veng Systems, Roslev, Denmark) in the chambers were also measured. Recovery tests (n = 40 for CO_2_ and n = 21 for CH_4_) were performed before, during, and after the experiments by infusing a known amount of pure CO_2_ or CH_4_ into the chambers and comparing it with the amount of gas measured by the system. Across chambers, average recovery values ± SD were 99.5 ± 1.45% for CO_2_ and 100.3 ± 2.37% for CH_4_. Recovery tests were used to correct the measured gas concentrations. The average of CH_4_ and CO_2_ recovery was used to correct O_2_ and H_2_. Throughout the experiment, the cows were assigned to the same specific respiration chamber for the first 48 h of gas measurements. For the latter 48 h of gas measurement, the cows were changed to the chamber along the diagonal to counteract eventual differences in background air composition.

### Microbiome and bioinformatics analysis

Rumen content samples for microbiome analyses were collected on d 9 at 14.00 h. Four grab samples from the dorsal, ventral, cranial, and caudal rumen were sampled through the rumen cannula and mixed. Approximately 50 g of mixed rumen content was immediately frozen at − 80 °C and subsequently freeze-dried and ground in a clean coffee grinder to a fine grind to ensure homogeneity of the sample. Weight of wet and dried samples were recorded and dried samples were stored at − 20 °C until analyzed. DNA was extracted from ~ 15 mg of dried rumen sample (actual weight recorded) with the NucleoSpin DNA stool kit (Macherey–Nagel, Düren, Germany) following the manufacturer’s directions and eluting in 150 µL of elution buffer. A negative control of water was included in the DNA extraction. Concentration of DNA was determined with the Qubit Broad range kit (Thermo Fisher Scientific, Wilmington, DE, USA).

Total bacteria and archaea as well as specific archaeal groups were quantified with qPCR using primers described in Table 2 (Sigma-Aldrich). Primer sequence, annealing temperature, and standard DNA for each pair of primers are listed in Table 2. Each reaction contained 5 µL of RealQ Plus 2× Master Mix, green (low ROX) (Amplicon III, Denmark), primers in final concentrations of 0.3 uM, 2 µL of template DNA, and nuclease-free water up to the final volume of 10 µL. The qPCR analysis was performed using a MicroAmp Optical 384-well reaction plate (Applied Biosystems) and an ABI ViiA7 real-time PCR system (Thermo Fisher Scientific) under the following run conditions; pretreatment of 2 min at 50 °C, followed by initial denaturation (15 min at 95 °C) and subsequently 40 cycles of denaturation for 15 s at 95 °C, annealing for 30 s at the temperature listed in Table 2, and 30 s at 72 °C for base extension. Melting curves were derived by increasing the temperature from 60 to 95 °C at a rate of 0.05 °C/s, recording continuously. All reactions were performed in triplicate and a no-template control was included in every run. Gene copies in the samples were calculated from a standard curve of fivefold serially diluted standard DNA with a known copy number and expressed as copies per g of rumen content (wet weight). The standards copy number was calculated from the DNA concentration, the number of target copies per genome and the genomes size^26^.

**Table 2:** Primers used for qPCR determination of microbial groups.

Target group		Sequence	Standard strain	Annealing temp.	References
*Methanobrevibacter*	F	TTTCGCCTAAGGATGGGTCT	*Methanobrevibacter ruminantium* M1 DSM 1093	60	^27^
*Methanobrevibacter*	R	CGATTTCTCACATTGCGGAG			
*Methanospheaera*	F	TAAGTCTTTGGTGAAAGCTT	*Methanosphaera stadtmanae* DSM 3091	60	^27^
*Methanospheaera*	R	GTTACTCACCGTCAAGAT			
*Methanomassiliicocales*	F	CAGCAGTCGCGAAAACTTC	*Methanomassiliicocus luminyensis B10*	60	^28^
*Methanomassiliicocales*	R	AACAACTTCTCTCCGGCAC			
Total archaea	F	AATTGGCGGGGGAGCAC	*Methanobrevibacter ruminantium* M1 DSM 1093	60	^29^
Total archaea	R	GGCCATGCACCWCCTCTC			
Total bacteria	F	CGGYCCAGACTCCTACGG	*Escherichia coli* K12	65	^30^
Total bacteria	R	TTACCGCGGCTGCTGGCAC			

Amplicon libraries covering the V3–V4 region of the 16S rRNA gene were prepared according to Noel et al.^31^ using universal primers Bac341F and Bac805R as recommended by Klindworth et al.^32^. Amplicon libraries were paired-end sequenced (2 × 300 bp) on the Illumina MiSeq platform (Illumina, San Diego, CA, USA). Raw microbiome sequence reads were deposited in the NCBI short-read archive database under BioProject ID: PRJNA906944 (https://www.ncbi.nlm.nih.gov/bioproject/PRJNA906944). Demultiplexed sequence reads were processed with QIIME2 v2022.2^33^. Briefly, primers were removed by base trimming, raw reads were quality filtered, denoised, and merged, PCR chimeras were removed and amplicon sequence variants (ASV) were inferred using the DADA2 v2022.2 plugin^34^ applying the following parameters: left-side trimming at base 17 for forward- and at base 21 for reverse reads (primer removal), right-side read truncation at base 280 for forward- and at base 262 for reverse reads (removing poor quality bases) and default parameter settings otherwise. A total of 5870 ASVs were detected. ASVs were taxonomically assigned using a 16S V3–V4 specific Naïve Bayes classifier trained on 99% similarity clustered 16S rRNA gene sequences extracted from the SILVA v138 reference database and trimmed to cover the V3–V4 region bound by the Bac341F and Bac805R primer pair. Microbial taxonomy of rumen microbiota used throughout the paper is in accordance with the taxonomy reported in the SILVA v138 database. For phylogenetic inference, ASVs were aligned with Mafft v7.310^35^, highly variable positions were masked, an unrooted phylogenetic tree was constructed with FastTree v2.1.10^36^ and rooted at the midpoint of the longest tip-to-tip distance.

### Laboratory analyses

Dry matter content of fresh feed and residue samples was determined by daily drying at 60 °C for 48 h^37^. Feed and digesta samples were freeze-dried and ground on a 1-mm screen prior to chemical analysis, except for a 0.5-mm screen used for analysis of starch (Ultra Centrifugal Mill ZM 200, Verder Scientific, Hann, Germany). Ash content was determined by combustion at 525 °C for 6 h. The content of N and C in feed and digesta samples were determined using a Vario Macrocube elemental analyzer (Elementar, Langenselbold, Germany). Crude protein was calculated as nitrogen × 6.25. Crude fat was determined by Soxhlet extraction with petroleum ether (Soxtec 2050, Foss Analytical, Hillerød, Denmark) after hydrolysis with HCl^38^. The concentration of neutral detergent fiber (aNDFom), acid detergent fiber (ADF), and acid detergent lignin (ADL) in the PMR and concentrate mixture were determined sequentially following ANKOM procedures^39^ in an ANKOM^2000^ Fiber Analyzer (ANKOM Technology, Macedon, New York, USA) using heat-stable α-amylase and sodium sulfite^40^. aNDFom in digesta samples and feces were also determined using heat-stable α-amylase and sodium sulfite in the ANKOM^2000^ Fiber Analyzer and reported as ash-free NDF. Starch was digested with heat-stable α-amylase and amyloglucosidase, and the reaction was subsequently assayed for glucose^41^ by using a YSI model 2900 analyzer (YSI Inc., Yellow Springs, OH). Content of TiO_2_ in digesta and feces samples was analyzed according to Myers et al.^42^, while content of Cr_2_O_3_ in digesta and feces was analyzed by oxidation to chromate and afterwards determined spectrophotometrically using Lamba 900 equipment (PerkinElmer Inc., Waltham, Massachusetts, USA^43^).

Concentrations of VFA were determined in stabilized rumen fluid after methanol-chloroform extraction with 2-ethylbutyrate as internal standard, using a GC (Trace 1310, Thermo Scientific, Germany) with split/splitless injector at 225 °C and a flame ionization detector at 250 °C. A 30 m × 0.53 mm × 1 µm HP-FFAP column (Agilent Technologies Inc., Wilmington, DE) was used with helium as carrier gas at 0.3405 atm. The GC oven was programmed to increase from 100 to 200 °C at 10 °C/min. Concentration of NH_3_ in ruminal fluid was measured using Randox Ammonia Kit-AM1015 and Cobas Mira Plus (Roche) after being diluted by phosphate buffer (100 mmol/L). l-lactate and glucose were analyzed using the immobilized glucose oxidase electrode technique^44^ (YSI 2900D, YSI Inc., Yellow Springs, USA).

Urine creatinine was determined according to standard procedures (Siemens Diagnostics® Clinical Methods for ADVIA 1800® Chemistry System; Siemens Medical Solutions, Tarrytown, New York, USA). Milk samples were analyzed for contents of fat, protein, lactose monohydrate, urea, and composition of fatty acids^45^ by mid-infrared reflection (MilkoScan™ 7 RM; Eurofins Steins Laboratorium A/S, Vejen, Denmark).

The concentrations of glucose, l-lactate, and urea in serum were measured by a spectrophotometric assay following the manufacturer’s guidelines (Siemens Medical Solutions, Tarrytown, New York, USA). Non-esterified fatty acids were determined using the Wako, NEFA C ACS-ACOD assay method. β-OH-butyrate was determined using a method involved oxamic acid in the media to inhibit lactate dehydrogenase followed by measurement of the absorbance at 340 nm due to the production of NADH^46^. All analyses were performed using an auto-analyzer, ADVIA 1800 ®Chemistry System (Siemens Medical Solutions, Tarrytown, New York, USA). Thyroxine in plasma was analyzed at the Central Veterinary Laboratory at University of Copenhagen, Denmark, and measured using the Immulite® 2000 immunoassay system (Immulite 2000, Siemens Healthineers, Erlangen, Germany). Bile acid, total protein, albumin, AST, g-GT, GLDH, and total bilirubin were analyzed in serum by Laboklin Laboratory for Clinical Diagnostics GmbH & Co. KG (Bad Kissingen, Germany) using the photometric method on a Roche Cobas® 8000 (Roche Diagnostics, Indianapolis, USA).

### Calculations and statistical analyses

Total dry matter intake was calculated based on amount of ingested PMR and concentrate mixture used for supplementation of iodoform inter-ruminally. Total water intake was calculated as the sum of measured ingested water and water intake from PMR and concentrate. Water excreted in feces was measured as the difference between total fecal flow and DM flow. Estimated excretion of water in milk was calculated as kg milk from which fat, protein, and lactose content were subtracted, ignoring an assumed similar mineral content between treatments, while estimated excretion of water in urine was calculated as total intake subtracted by water excreted in feces and estimated excretion in milk, ignoring an assumed similar and assumed insignificant evaporation of water from the animals.

Gross energy contents in PMR and concentrate were calculated according to NorFor^24^. Milk yield was converted to ECM (3.140 MJ/kg), according to Sjaunja et al.^47^:

$${\text{ECM}} = {\text{milk yield }} \times \, \left[ {\left( {{38}.{3 } \times {\text{ fat }} + { 24}.{2 } \times {\text{ protein }} + { 15}.{71 } \times {\text{ lactose }} + { 2}0.{7}} \right)/{3,14}0} \right],$$

with ECM and milk yield in kilograms; fat, protein, and lactose monohydrate in grams per kilogram. Content of total fatty acids in milk was calculated according to Schwarz et al.^45^ as fat-% × 0.95. The respiratory coefficient (RQ) was calculated as the ratio between CO_2_ produced and oxygen consumed (L/L). Energy balance was calculated as difference between net energy intake (MJ/day) and energy excretion in milk (MJ/day).

Duodenal, ileal and fecal DM flows were averaged across markers, assuming concentrations in pooled digesta samples were representative for the average daily flow of digesta. The flows of OM, NDF, crude protein (CP), carbon (C), and starch in duodenum, ileum, and large intestine were calculated from the DM flow and their respective concentrations in each section of the digestive tract. Intake and flows of DM, OM, NDF, CP, C, and starch at duodenum, ileum, and feces were used to calculate apparent digestibility of the nutrients in the different sections of the gastrointestinal tract. Redox potential was not converted relative to a standard hydrogen electrode.

Gas exchange was measured as flows at standard temperature and pressure (STP (0 °C (273.15 K) and 101.325 kPa)). The calculated production or use of gases in L/day was converted to g/day using the density of each gas at STP which were 0.716, 1.963, 0.0899, and 1.428 (L/g) for CH_4_, CO_2_, H_2_, and O_2_, respectively. The hourly emissions was calculated as the sum of gas produced within the clock hour. Gas emissions over an hour shift were divided according to percent of time in the respective clock hours. Data was deleted when chambers were open and cows were milked and fed. Furthermore, it was assumed that the cows had a similar gas production for deleted minutes as the average for all minutes for each measuring period.

Observations of all variables were averaged within cow and period. There were 15 observations in total as the cow given 1080 mg/day of iodoform was omitted from the used data.

Statistical analyses were conducted in R 4.1.2 (R Core Team, 2021). The effect of treatment on the various animal responses was analyzed with the following linear mixed model fitted with REML and the “lmer” function from the “lme4” package^48^:

$${\text{Y}}_{{{\text{tpc}}}} = \upmu + \alpha_{{\text{t}}} + \upgamma_{{\text{p}}} + {\text{ A}}_{{\text{c}}} + \, \upvarepsilon_{{{\text{tpc}}}} ,$$

where Y_tpc_ is the dependent response variable, μ is the overall mean, α is the fixed effect of treatment (t = 0 mg/day, 320 mg/day, 640 mg/day, or 800 mg/day), γ is the fixed effect of period (p = 1 to 4), A is the random effect of cow (c = 1 to 4), and ɛ_tpc_ is the random residual error assumed to be independent with constant variance and normally distributed. The data was tested for normality of the residuals by evaluating the QQ-plots constructed in R and using the Shapiro–Wilk test. Homogeneity of the variance was tested by evaluating plots of residuals and using Bartlett’s test. Data is presented in tables as estimated marginal means (EMS) and standard error of mean. They were obtained using the “emmeans” package.

Hourly CH_4_ and H_2_ emissions were analyzed with the following model:

$${\text{Y}}_{{{\text{thpc}}}} = \upmu + \upalpha_{{\text{t}}} + \uptau_{{\text{h}}} + \upalpha_{{\text{t}}} \times \tau_{{\text{h}}} + \upgamma_{{\text{p}}} + {\text{ A}}_{{\text{c}}} + \, \upvarepsilon_{{{\text{thpc}}}} ,$$

where Y_thpc_ is the dependent response variable, μ is the overall mean, α is effect of treatment (t = 0, 320, 640 or 800 iodoform/kg DM), τ is the fixed effect of hour (h = 0 to 23), α_t_ × τ_h_ is the interaction, γ is the effect of period (p = 1-4  3), A is the effect of cow (c = 1 to 4), and ɛ_thpc_ is the random residual error assumed to be independent with constant variance and normally distributed. Data was analyzed using a first-order autoregressive covariance structure with heterogeneous variance (AR1).

To test the linear and quadratic effects of treatment the following model was applied:

$${\text{Y}}_{{{\text{tpc}}}} = \upmu + \upalpha \times {\text{ D}}_{{{\text{tpc}}}} + \upbeta \times \left( {{\text{D}}_{{{\text{tpc}}}} } \right)^{{2}}_{{}} + \upgamma_{{\text{p}}} + {\text{ A}}_{{\text{c}}} + \, \upvarepsilon_{{{\text{tpc}}}} ,$$

where D_tpc_ is doses determined by treatment, period and cow, β is the quadratic effect and the linear effect is given by the parameter α in the model with β set to zero. The significance of these effects was tested using a F-test, based on Kenward-Roger approximation, from the “pbkrtest” package.

Two-way analysis of variance (ANOVA) was used to compute the P-values for the fixed effects. Differences between EMS were evaluated using Tukey’s method for comparison. Statistical significance was declared when *P* ≤ 0.05 and statistical tendencies were declared when 0.05 < *P* ≤ 0.10.

The feature table, sample data, tree, and taxonomic classifications were imported into R as phyloseq object^49^ for subsequent microbial data analyses. If not stated differently, subsequent analyses were conducted using prevalence filtered, pruned, and rarefied ASV data.

Top 10 most abundant bacterial families across all samples and grouped by iodoform treatment were identified and visualized as heat maps using the ampvis2 package v2.7.11^50^. Alpha-diversity metrics (observed richness, Shannon’s diversity index, and Faith’s phylogenetic diversity index) were computed with phyloseq and differences in alpha diversity between treatment groups were tested using a Kruskal–Wallis rank sum test. Differences in beta diversity and rumen microbiota composition were investigated using the vegan v2.5-7 package^51^. Briefly, Bray–Curtis dissimilarity distances were estimated with phyloseq. Homogeneity of group dispersions (variance) was analyzed using the *betadisper* function in vegan, differences in microbial composition between treatment groups were investigated by Principal Coordinate Analysis (PCoA) on Bray–Curtis dissimilarities and group significances were computed by permutational multivariate analysis of variance (PERMANOVA) applying the *adonis* function with 9999 permutations and default settings otherwise. PCoA ordination plots were generated with the ggplot2 package v3.3.5^52^. Microbial compositions were considered significantly different at *P* ≤ 0.05. Differential abundant ASVs were identified using a negative binomial generalized linear model approach implemented in the DESeq2 package v1.3.4^53^ with pruned and prevalence filtered (not rarefied) ASV counts present in at least 10% of the samples and collapsed to genus level and response variable and iodoform treatment as fixed effect. Briefly, ASV counts were normalized by variance-stabilizing transformation, size factors for each ASV were estimated applying a median-ratio-method, dispersions of ASV counts were computed and a negative binomial WaldTest was performed^53^. Pairwise comparisons were computed for 320 vs. 0, 640 vs. 0 and 800 vs. 0. P-values were adjusted using the Benjamini–Hochberg procedure^54^ and ASVs were considered differentially abundant when FDR ≤ 0.05 and log2 fold changes ≥ 2 or ≤  − 2.

### Ethics approval and consent to participate

The experiment complied with the guidelines set out by the Danish Ministry of Environment and Food with respect to animal experimentation and care of animals under study (Act No. 2028, 2020).

## Results

### Feed intake, milk yield, and composition of milk fatty acids

Dry matter intake decreased linearly with increasing dose of iodoform. The decrease was approximately 32 and 48% for 640 and 800 mg/day, respectively, compared to 0 mg/day (Table 3). Due to the reducing effect on DMI, the roughage share of total DMI varied slightly between treatments. Hence, roughage shares ranged from 50.7–51.2, 50.0–51.3, 48.0–50.7, to 47.4–50.1% for 0, 320, 640, and 800 mg/day, respectively. Similarly, a linear decrease was observed for milk yield and daily amounts of produced lactose, milk fat and milk protein with increasing dose of iodoform; however, the depression in milk yield (ECM) was less pronounced (15 and 33% for 640 and 800 mg/day, respectively, compared to 0 mg/day) than the depression in DMI. A linear increase in fat percentage and decrease in protein percentage were observed with increasing dose of iodoform. Decreases in proportion of medium-chain fatty acids (MCFA), but higher proportions of long-chain fatty acids (LCFA) of total fatty acids (FA) and mono-unsaturated FA at the expense of saturated FA were also found with increasing iodoform dose.

**Table 3:** Dry matter intake (DMI), milk production expressed in energy corrected milk (ECM), and proportions of fatty acids (FA) as % of total FA from dairy cows supplemented with four different levels of iodoform (0, 320, 640, and 800 mg/day) intra-ruminally twice daily.

	Iodoform supplementation				SEM	P-values		
	0 mg/day	320 mg/day	640 mg/day	800 mg/day		Treatment	Linear effect	Quadratic effect
DMI in chambers, kg/day	22.5^a^	20.3^ab^	15.4^ab^	11.7^b^	2.46	0.04	< 0.01	0.32
Net energy intake, MJ/day	151.7^a^	136.8^ab^	103.8^ab^	79.2^b^	16.4	0.04	< 0.01	0.15
Milk, kg	31.7^a^	30.8^ab^	26.3^ab^	20.2^b^	3.26	0.04	0.01	0.12
ECM, kg/day	32.6^a^	32.1^ab^	27.7^ab^	21.7^b^	2.89	0.05	0.02	0.10
Energy balance^1^, MJ/MJ	49.3	36.4	16.9	9.51	9.06	0.03	< 0.01	0.62
Fat, kg/day	1.28	1.29	1.14	0.91	0.105	0.06	0.03	0.08
Protein, kg/day	1.19	1.15	0.957	0.732	0.118	0.06	0.01	0.17
Lactose, kg/day	1.52^a^	1.44^a^	1.25^ab^	0.961^b^	0.137	0.02	< 0.01	0.12
Fat, %	4.11	4.29	4.40	4.49	0.336	0.26	0.03	0.91
Protein, %	3.81	3.75	3.64	3.59	0.285	0.12	0.02	0.50
Lactose, %	4.78	4.82	4.78	4.69	0.040	0.24	0.69	0.78
Urea, mM	0.492	0.493	0.480	0.429	0.0386	0.06	0.01	0.36
% of FA^2^								
C14:0	12.9^a^	12.3^ab^	11.5^ab^	10.0^b^	0.592	0.03	< 0.01	0.32
C16:0	32.1	31.0	29.8	27.1	1.62	0.15	0.03	0.44
C18:0	8.84	9.67	9.57	10.85	0.864	0.21	0.07	0.81
C18:1 (*cis-9*)	21.3	21.5	24.2	28.4	1.85	0.06	0.03	0.16
SCFA	12.6	12.9	12.1	10.7	0.915	0.33	0.16	0.24
MCFA	50.9^a^	48.8^ab^	46.6^ab^	41.5^b^	1.93	0.03	< 0.01	0.29
LCFA	36.5^b^	38.3^ab^	41.3^ab^	47.8^a^	2.45	0.04	0.01	0.23
Saturated FA	74.0	73.0	70.2	65.3	2.34	0.08	0.02	0.21
Mono-unsaturated FA	25.2	25.0	27.5	31.6	1.73	0.07	0.04	0.12
Poly-unsaturated FA	3.63	3.87	3.68	4.00	0.246	0.64	0.46	0.97
Trans-unsaturated FA	2.83	3.23	2.85	3.65	0.461	0.47	0.39	0.85
De novo FA^3^	28.6	28.4	27.3	23.8	1.37	0.10	0.05	0.19
Mixed FA^3^	35.1	34.3	33.3	29.9	1.29	0.07	0.03	0.24
Preformed FA^3^	32.0^b^	33.0^ab^	35.6^ab^	42.9^a^	2.48	0.04	0.03	0.16

^ab^Values within the same line with different superscripts differ (P < 0.05).

^1^Calculated as difference between net energy intake (MJ/d) and energy in milk (MJ/d).

^2^Fatty acids calculated as fat-% × 0.95, according to Schwarz et al.^45^.

^3^Sum of de novo, mixed, and preformed FA do not add up to 100% as FA is calculated as fat-% × 0.95.

### Gas exchange

**Figure 1:**
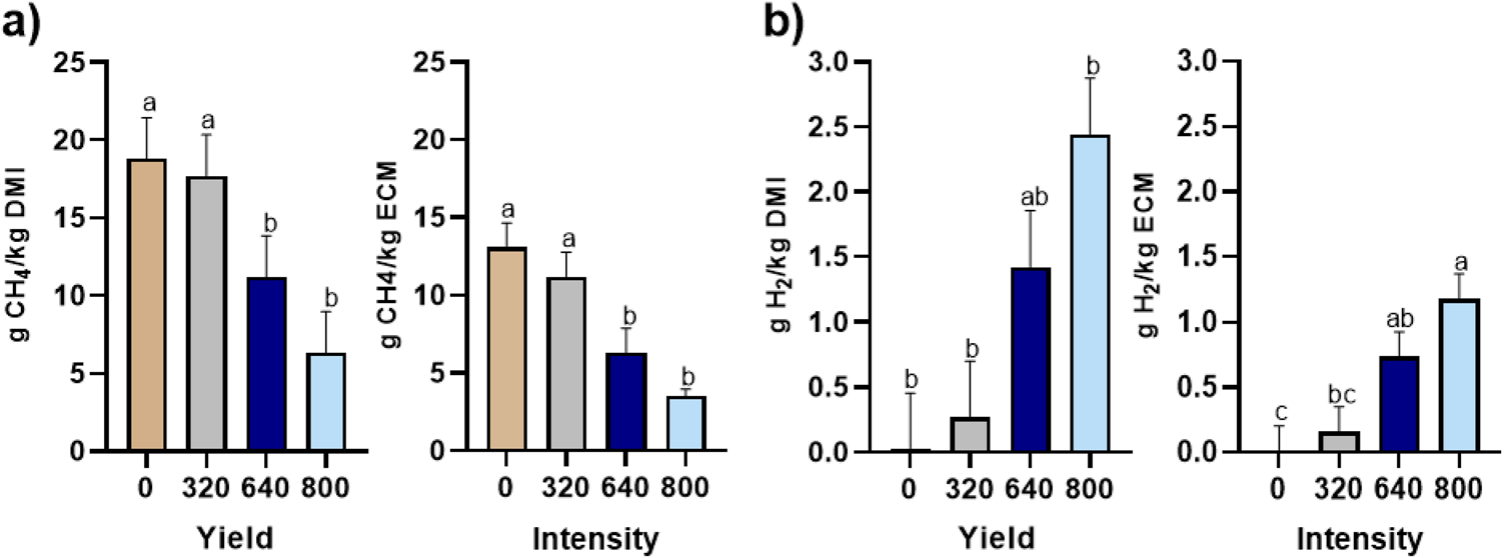
(**a**) Estimated marginal means of methane (CH_4_) yield (g CH_4_/kg dry matter intake (DMI)), treatment P < 0.01; linear P < 0.01; quadratic P ≤ 0.01 and intensity (g CH_4_/kg energy corrected milk (ECM)), treatment P < 0.01; linear P < 0.001; quadratic P = 0.03 (**b**) and hydrogen (H_2_) yield (g H_2_/kg DMI), treatment P < 0.01; linear P < 0.01; quadratic P = 0.06 and intensity (g H_2_/kg ECM), treatment P < 0.01; linear P < 0.001; quadratic P = 0.05 of dairy cows supplemented with four different levels of iodoform (0, 320, 640, and 800 mg/day) intra-ruminally twice daily (6:30 h and 16:30 h).

**Figure 2:**
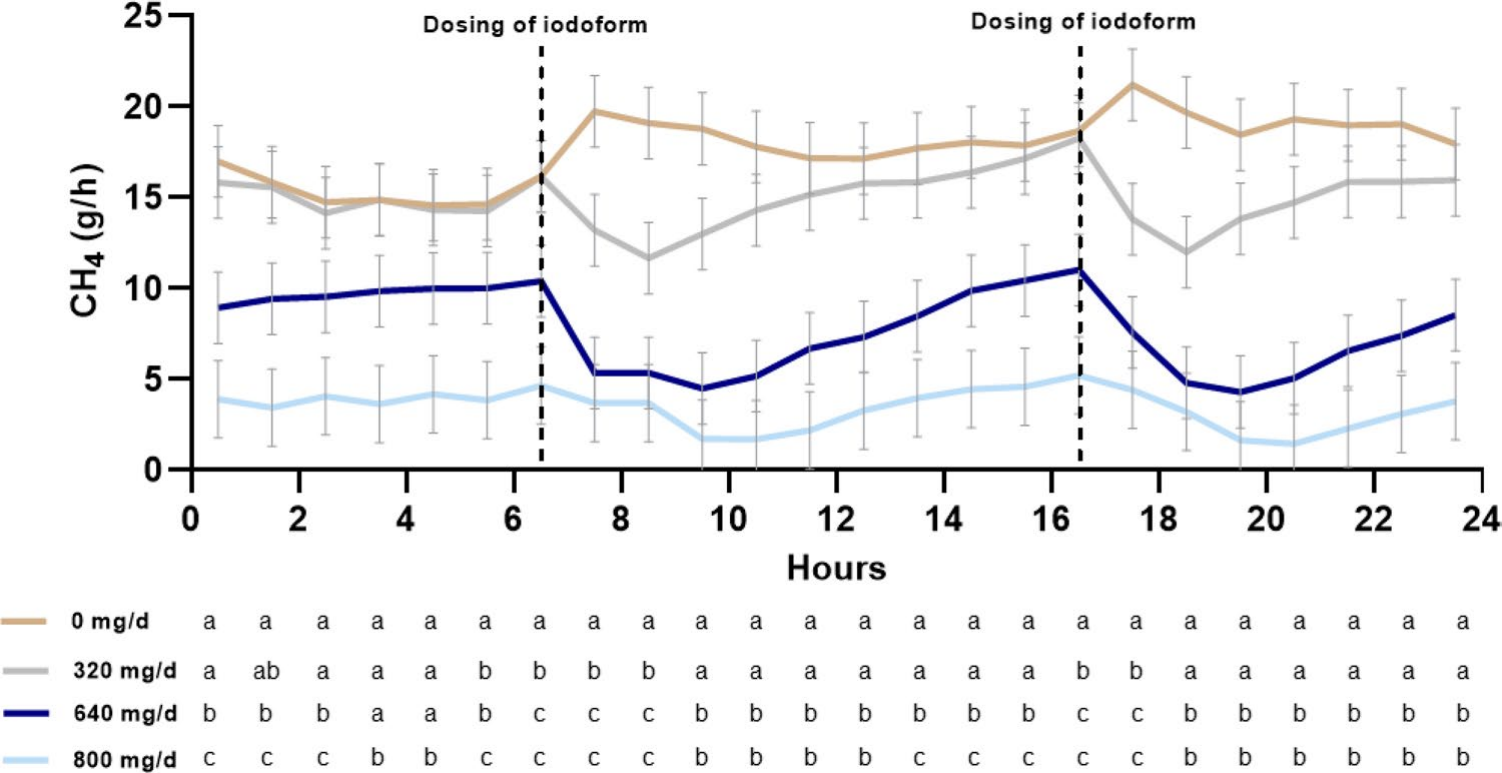
Estimated marginal means of hourly methane (CH_4_) emission from dairy cows supplemented iodoform intra-ruminally twice daily. Stippled vertical lines indicate time of iodoform dosing. Each time point represents the average methane production for 1 h before and after that time (i.e., time point 0630 h represents the average gas production from 0600 to 0700 h). Below the figure, different letters for different treatments at the same time point are significantly different at P < 0.05.

Daily CH_4_ emission decreased linearly with increasing dose of iodoform from 423 g/day at 0 mg/day to 94.9 g/day at 800 mg/day. This decrease was accompanied with a dramatic increase in H_2_ emission (Table 4). Methane yield, expressed as g CH_4_/kg DMI, decreased with increasing dose of iodoform. Hence, CH_4_ yield decreased by 40 and 66% at iodoform supplementation of 640 and 800 mg/day, respectively, compared to 0 mg/day (Fig. 1a). Simultaneously, H_2_ yield (g H_2_/kg DMI) increased 125-fold (Fig. 1b) at 800 mg/day compared to 0 mg/day. A similar pattern was observed for CH_4_ intensity (g CH_4_/kg ECM) and H_2_ intensity (g H_2_/kg ECM). Methane intensity decreased by 73% on 800 mg/day compared to 0 mg/day, while H_2_ intensity increased 88-fold. There were substantial diurnal variations in emissions between the treatments. Immediately after feeding, CH_4_ emission increased in cows supplemented 0 mg/day, whereas intra-ruminal dosing of iodoform resulted in a depression of CH_4_ emission right after feeding (Fig. 2).

**Table 4:** Daily gas exchange and rumen headspace gas ratios in dairy cows supplemented with four different levels of iodoform (0, 320, 640, and 800 mg/day) intra-ruminally twice daily.

	Iodoform supplementation				SEM	P-values		
	0 mg/day	320 mg/day	640 mg/day	800 mg/day		Treatment	Linear effect	Quadratic effect
Gas exchange, g/day								
CH_4_	423^a^	358^ab^	187^bc^	94.9^c^	54.5	< 0.01	< 0.001	0.19
CO_2_	14865^a^	13626^a^	11117^ab^	8831^b^	1395	0.03	< 0.01	0.26
O_2_	9719^a^	9059^ab^	7856^ab^	6567^b^	786	0.03	< 0.01	0.27
H_2_	0.443^c^	5.014^bc^	19.4^ab^	23.8^a^	4.12	< 0.01	< 0.001	0.31
Respiration coefficient, L/L	1.09^a^	1.09^a^	1.02^ab^	0.96^b^	0.027	0.01	< 0.01	0.03
Headspace samples, ppm/ppm								
CH_4_:CO_2_ ratio	0.265^a^	0.216^ab^	0.168^bc^	0.083^c^	0.032	< 0.01	< 0.001	0.19
CH_4_:H_2_ ratio	–*	–*	2.21	1.17	0.018	0.03	–	–

^a–c^Values within the same line with different superscripts differ (P < 0.05).

*Values for H_2_ were below the detection level (10,000 ppm).

The treatment was stopped on day 11 in period 1 for the cow that initially received a supplementation dose of 1080 mg/day of iodoform, due to a large depression of DMI. After cessation of the treatment, DMI and gaseous emissions began to return to pre-treatment levels. After 3 days, DMI had doubled and the daily production of CH_4_ increased by a factor of 22 (Supplementary Table 1).

### Water intake and excretion

Total water intake decreased linearly with increasing dose of iodoform and lower DMI (Supplementary Table 2). A linear increase in proportion of water intake excreted in milk was also found with increasing iodoform dose, while no effects were found on the proportion excreted in feces and urine.

### Nutrient digestibility in the gastrointestinal tract

In the rumen, linear decreases in digestibility of DM, OM, C, and CP were seen with increasing dose of iodoform (Table 5). A tendency to decreased ruminal digestibility of starch was also found, while digestibility of NDF was unaffected by treatments. However, in contrast to this, there was a compensatory increase in digestibility in the small intestine for DM, OM, C, and CP with increasing dose of iodoform, and hence total tract digestibility were unaffected by iodoform supplementation since iodoform supplementation did not affect large intestine nutrient digestibility.

**Table 5:** Apparent digestibility of dry matter (DM), organic matter (OM), neutral detergent fiber (NDF), carbon (C), crude protein (CP), and starch in dairy cows supplemented with four different levels of iodoform (0, 320, 640, and 800 mg/day) intra-ruminally twice daily.

	Iodoform supplementation				SEM	P-values		
	0 mg/day	320 mg/day	640 mg/day	800 mg/day		Treatment	Linear effect	Quadratic effect
Intake during sampling period, kg/day								
DM	23.8^a^	22.5^ab^	17.9^ab^	12.9^b^	2.60	0.03	< 0.01	0.14
OM	22.0^a^	20.8^ab^	16.5^ab^	11.9^b^	2.40	0.03	< 0.01	0.14
NDF	7.55^a^	7.13^ab^	5.68^ab^	4.10^b^	0.818	0.03	< 0.01	0.14
C	9.98^a^	9.45^ab^	7.52^ab^	5.42^b^	1.09	0.03	< 0.01	0.14
CP	3.84^a^	3.63^ab^	2.90^ab^	2.10^b^	0.414	0.03	< 0.01	0.14
Starch	3.63^a^	3.43^ab^	2.73^ab^	1.98^b^	0.393	0.03	< 0.01	0.14
Apparent ruminal digestibility, g/kg								
DM	371	322	284	245	37.6	0.11	< 0.01	0.87
OM	468	432	402	383	27.3	0.14	0.01	0.99
NDF	655	694	665	656	38.1	0.76	0.95	0.32
C	424	367	341	325	29.1	0.11	< 0.01	0.59
CP	− 109	− 229	− 248	− 300	56.3	0.10	0.01	0.52
Starch	865	861	805	806	31.4	0.32	0.07	0.66
Apparent small intestinal digestibility, g/kg								
DM	515^b^	586^b^	602^ab^	628^a^	23.4	0.02	< 0.01	0.32
OM	483^b^	557^ab^	574^ab^	595^a^	25.7	0.03	< 0.01	0.31
C	522^a^	604^ab^	617^b^	630^b^	22.2	0.02	< 0.01	0.13
CP	713^b^	755^ab^	756^a^	761^a^	10.2	0.03	0.01	0.10
Starch	867	895	902	930	33.9	0.50	0.17	0.92
Apparent large intestinal digestibility, g/kg								
DM	161	85.0	103	107	42.8	0.29	0.22	0.15
OM	133	44.1	72.1	80.5	43.2	0.23	0.28	0.10
NDF	179	30.1	47.6	115	57.0	0.09	0.25	0.02
C	122	32.1	50.0	74.3	40.9	0.21	0.28	0.07
CP	89.8	45.1	45.2	57.8	49.5	0.77	0.38	0.47
Starch	− 30.7	− 61.9	55.3	− 99.1	70.1	0.43	0.82	0.76
Apparent total tract digestibility, g/kg								
DM	748	745	746	749	11.7	0.98	0.99	0.71
OM	765	761	766	770	11.3	0.91	0.72	0.54
NDF	663	656	669	692	15.2	0.34	0.20	0.18
C	762	758	763	768	12.4	0.90	0.66	0.57
CP	714	713	709	707	17.6	0.98	0.60	0.89
Starch	985	985	984	986	3.40	0.98	0.86	0.91

^ab^Values within the same line with different superscripts differ (P < 0.05).

### Rumen fermentation pattern

With increasing dose of iodoform, treatment and linear decreasing effects were observed on rumen concentrations of total VFA, L-lactate, NH_3_, and redox potential (Table 6). Associated with the lower VFA concentration, increases in ruminal pH were also seen with increasing iodoform doses. Composition of VFA was also affected by increasing iodoform dose, where a linear decrease in the percentage of acetate and increases in butyrate, isobutyrate, and isovalerate were observed.

**Table 6:** Ventral ruminal pH, concentration of total VFA, proportion of individual VFA as % of total VFA and dorsal ruminal redox potential of dairy cows supplemented with four different levels of iodoform (0, 320, 640, and 800 mg/day) intra-ruminally twice daily.

	Iodoform supplementation				SEM	P-values		
	0 mg/day	320 mg/day	640 mg/day	800 mg/day		Treatment	Linear effect	Quadratic effect
pH	6.02^b^	6.04^b^	6.48^a^	6.66^a^	0.106	< 0.001	< 0.001	0.04
Redox, mV	− 251^a^	− 274^a^	− 364^ab^	− 426^b^	37.5	< 0.01	< 0.01	0.17
Total VFA, mM	136^a^	135^a^	115^ab^	102^b^	6.49	0.01	< 0.01	0.05
% of total VFA								
Acetate	57.9	56.7	55.2	54.2	1.33	0.18	0.02	0.82
Propionate	24.8	24.0	23.4	24.8	1.09	0.69	0.63	0.39
Butyrate	13.4^b^	14.9^ab^	16.2^a^	14.9^ab^	0.669	0.03	0.03	0.12
Isobutyrate	0.509^b^	0.548^b^	0.659^ab^	0.788^a^	0.0904	< 0.01	< 0.01	0.07
Valerate	1.71	1.74	1.81	1.85	0.179	0.49	0.10	0.72
Isovalerate	1.08^c^	1.45^bc^	1.95^b^	3.01^a^	0.224	< 0.001	< 0.001	0.05
Caproate	0.573	0.670	0.766	0.453	0.133	0.18	0.91	0.13
NH_3_, mM	5.50	5.56	4.02	2.76	0.794	0.07	0.02	0.13
Glucose, mM	2.32	2.19	1.39	0.864	0.209	< 0.01	< 0.001	0.02
l-Lactate, mM	2.93	2.46	0.678	0.530	0.694	0.06	< 0.01	0.62

^ab^Values within the same line with different superscripts differ (P < 0.05).

### Rumen microbiota

Amplicon sequencing of the microbial rumen community targeting the V3–V4 region of the 16S rRNA gene yielded a mean read number of 43,221 (excluding negative controls) merged and denoised sequence reads, ranging from 36,465 to 50,094 reads per sample. The negative control samples only contained 10 and 13 merged and denoised sequence reads, respectively, confirming the validity of the sequencing procedure. A total of 5870 ASVs were inferred ranging from 1131 to 1438 ASVs per sample (mean: 1232) before filtration, of which 2742 ASVs passed filtration procedures (per sample range 950 to 1248; mean 1081). Rarefaction curves leveled off at around 20,000 reads indicating that sample rarefication at 35,200 reads was sufficient to capture. The community diversity (not shown). The identity of top ten most abundant families across all the samples are shown in a heat map grouped by treatment (Fig. S1 Supplementary).

**Figure 3:**
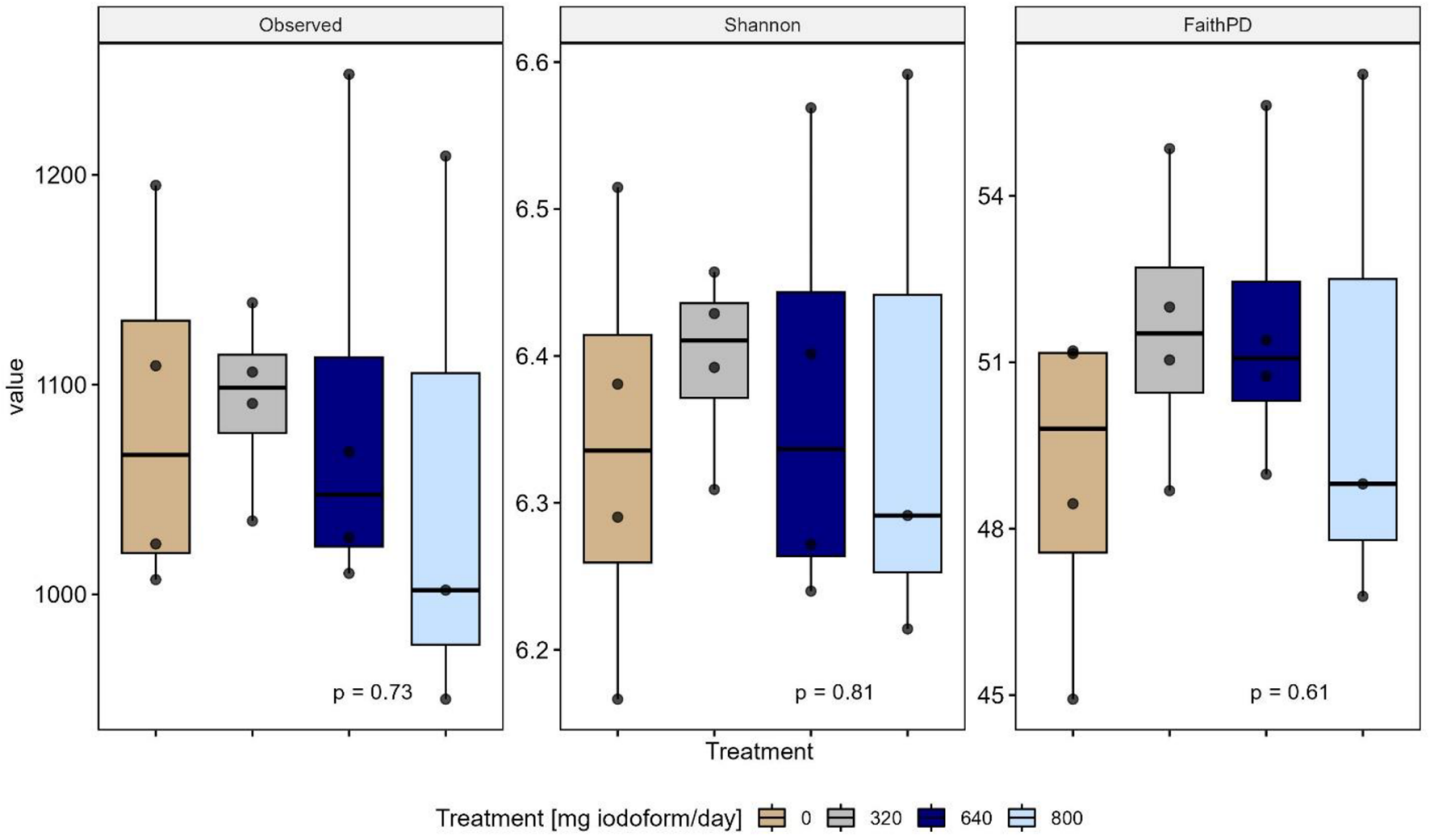
Alpha diversity estimates for observed, Shannon’s, and Faith’s Phylogenic alpha diversity metrics with samples grouped by iodoform treatment. Alpha diversities were estimated for prevalence filtered, pruned and rarefied ASV counts. Treatment differences were estimated using a Kruskal–Wallis rank sum test. P-values ≤ 0.05 are considered significant.

**Figure 4:**
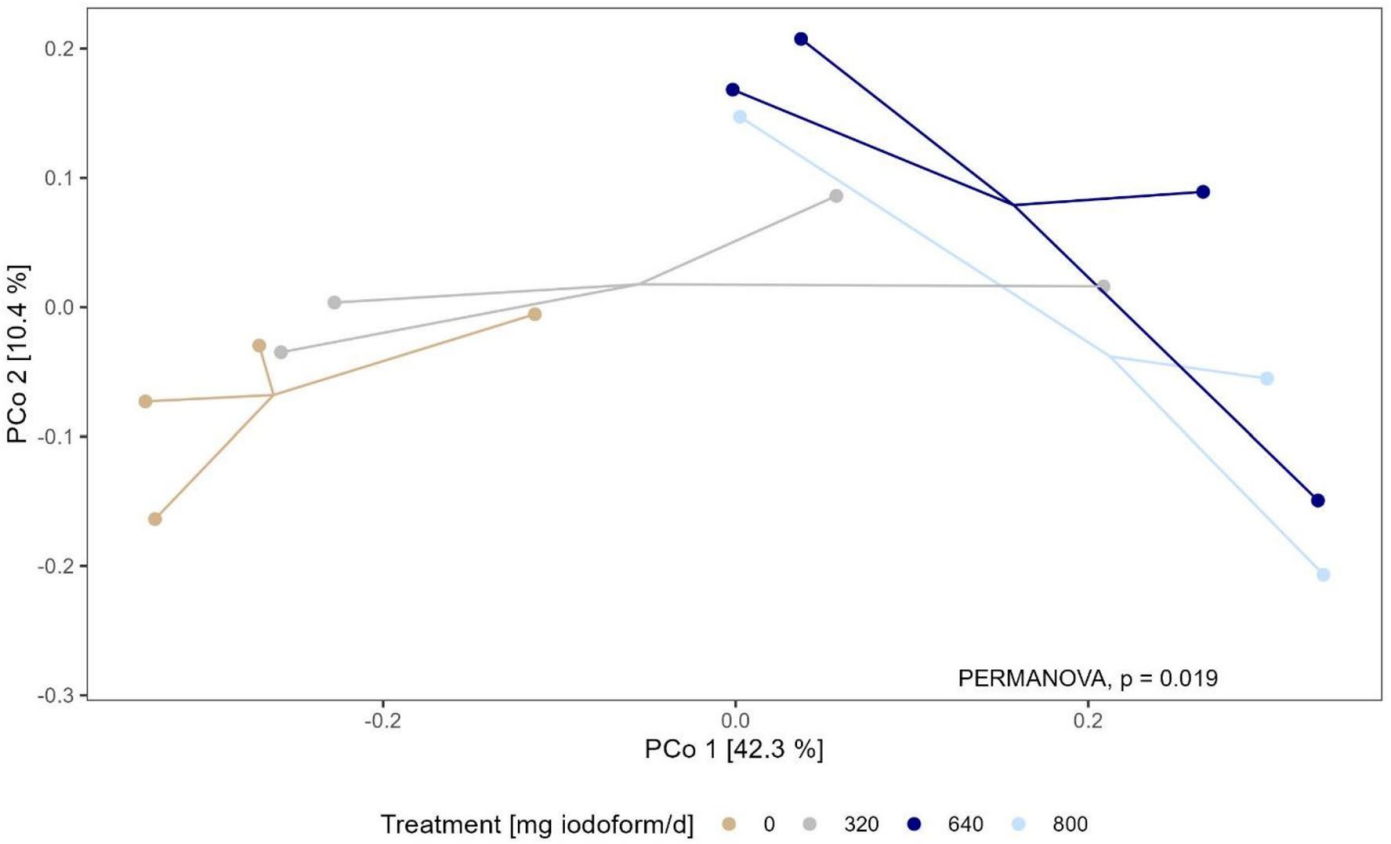
Principal coordinate (PCo) ordination plot based on Bray–Curtis distances representing iodoform treatment effect on rumen microbial community composition on PCo 1 and PCo 2. Bray–Curtis distances were estimated for prevalence filtered, pruned and rarefied ASV counts and colored based on iodoform treatment group: 0 mg/day, 320 mg/day, 640 mg/day, 800 mg/day. Total variance explained by each PCo is stated in parenthesis in the axis’s labels. Overall differences in treatment group centroids (group means of Bray–Curtis distances) were estimated by PERMANOVA. P-values ≤ 0.05 are considered significant.

Alpha diversity metrics estimated on microbial richness (observed richness: *P* = 0.73), on microbial richness and evenness (Shannon’s diversity Index: *P* = 0.81) or on phylogenetic distances between detected microbiota (Faith’s phylogenetic diversity index: *P* = 0.61) did not differ between treatment groups revealing that iodoform, irrespective of dose, did not affect rumen microbiota alpha diversity (Fig. 3). Moreover, beta diversity analysis revealed no significant differences between groups in terms of dispersion (homogeneity of variance) around treatment centroids (F = 3.62; *P* = 0.06). However, comparison between groups showed that iodoform treatment had a significant impact on microbial community composition (F = 2.15, *P* = 0.02), with iodoform treatment explaining 37% of the total variance. Differences in microbial composition are visualized by a PCoA ordination plot (Fig. 4) based on Bray–Curtis distances with PCo1 and PCo2 explaining 42.3% and 10.4% of the total variance, respectively.

**Figure 5:**
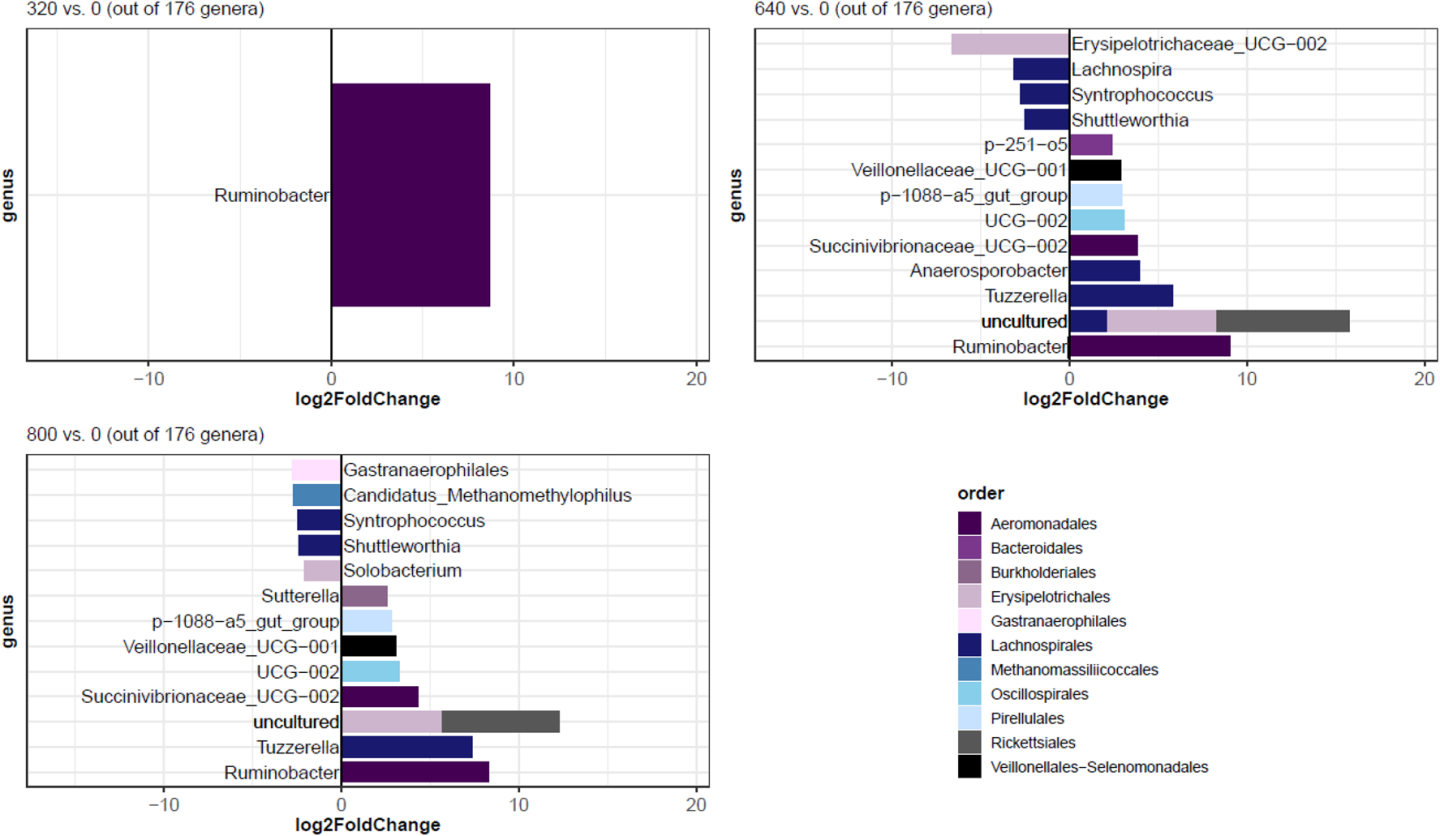
Differential abundant genera identified for the following iodoform treatment comparisons: 320 mg/day vs. 0 mg/day; 640 mg/day vs. 0 mg/day, 800 mg/day vs. 0 mg/day. Differential abundance analysis was conducted for prevalence filtered and pruned ASV counts present in at least 10% of the samples and collapsed to genus level. Color codes applied represent order level assignment. Negative log2 fold changes refer to a reduction in abundance in the iodoform treated group, whereas positive fold changes refer to an increase in abundance in the iodoform treated group. Multicolored bars represent genera with identical genus name that belong to different orders, this is only the case for uncultured genera. Statistical reports are found in Table S3.

In total, 19 genera were identified as differentially abundant when compared with the group not receiving iodoform (Fig. 5, Table S3). The genus *Ruminobacter* showed the largest increase in abundance (log2 fold changes for: 320 vs. 0 = 8.7; 640 vs. 0 = 9.1; 800 vs. 0 = 8.3) and was increased in all three iodoform treated groups. In addition, nine genera showed comparable abundance patterns both in the 640 and 800 mg dose group (seven genera increased in abundance, two decreased in abundance). The remaining nine genera detected as differentially abundant were dose specific (Fig. 5). The largest decrease in abundance was detected for the genus *Erysipelotrichaceae_UCG-002* (log2 fold change for 640 vs. 0 =  − 6.7) but changes in *Erysipelotrichaceae_UCG-002* abundance were limited to cows receiving 640 mg iodoform/day.

The number of total methanogens, *Methanobrevibacter*, and *Methanosphaera* (log10 copies/g rumen content) determined by qPCR decreased linearly with increasing dose of iodoform (Table 7). Total methanogen copies decreased from log10 8.62 to log10 7.68 with the 800 mg dose, *Methanobrevibacter* copies decreased from log10 8.2 to log10 6.58 and *Methanosphaera* decreased from log10 9.06 to log10 6.88 with the 800 mg dose. The *Methanomassiliicoccales* had a tendency for a linear response but were not significantly affected by iodoform treatments and showed a small numerical decrease from log10 7.3 to log10 6.88 at the highest iodoform dose. The total bacteria copy numbers (log10 copies/g rumen content) were unaffected by iodoform doses.

**Table 7:** Counts of methanogens and bacteria in rumen content from dairy cows supplemented with four different levels of iodoform (0, 320, 640, and 800 mg/day) intra-ruminally twice daily.

	Iodoform supplementation				SEM	P-values		
	0 mg/day	320 mg/day	640 mg/day	800 mg/day		Treatment	Linear effect	Quadratic effect
Total methanogens, log10 copies/g	8.62^a^	8.46^a^	8.31^ab^	7.68^b^	0.167	0.02	0.01	0.16
*Methanobrevibacter*	8.20^a^	7.96^a^	7.29^ab^	6.58^b^	0.286	0.01	< 0.01	0.11
*Methanosphaera*	9.06^a^	8.89^a^	8.68^ab^	7.45^b^	0.294	0.02	0.03	0.12
*Methanomassiliicoccales*	7.30	7.16	7.16	6.88	0.128	0.17	0.06	0.62
Total bacteria, log10 copies/g	9.17	9.06	9.07	9.08	0.0446	0.22	0.15	0.16

^ab^Values within the same line with different superscripts differ (P < 0.05).

In the present study, the adaptation period was relatively short (7 day). Figure S2 Supplementary shows the microbial composition of the cows, based on Bray Curtis distances. The samples originating from the cow that received 1080 mg/day in the first period are represented by star-shaped symbols. The red star in Fig. S2 indicates 1080 mg/day, while the brown and tan star(s) represent samples from the same cow in the following period and in the third and fourth periods, respectively, where no iodoform was supplemented. From the figure, it can be observed for the cow receiving the highest dose (1080 mg/day) in the first period that the microbial composition in the following period resembled the microbial composition  for the third and fourth periods (0 mg iodoform/day), indicating it was stable after one period of ceasing the treatment.

### Metabolic and health indicators

Serum levels of NEFA, total bilirubin, and urea increased linearly with increasing dose of iodoform, whereas concentrations of T4 in plasma decreased (Table 8). Values for all liver health biomarkers were within the normal reference range, except for g-GT and GLDH, where all treatments, except 800 mg/day, had levels above the upper reference value. Creatinine levels in urine increased linearly with increasing dose of iodoform.

**Table 8:** Indicators of metabolic and health status of dairy cows in urine and serum (unless stated otherwise), supplemented with four different levels of iodoform (0, 320, 640, and 800 mg/day) intra-ruminally twice daily.

	Iodoform supplementation					P-values			Reference value
	0 mg/day	320 mg/day	640 mg/day	800 mg/day	SEM	Treatment	Linear effect	Quadratic effect	
Urinal indicators of metabolic status									
Creatinine, µM	5387^b^	5590^b^	6983^ab^	9179^a^	7.78	0.02	< 0.01	0.06	–
pH	8.02^b^	8.05^ab^	8.06^a^	8.04^ab^	0.021	0.04	0.06	0.02	–
Serum indicators of metabolic status									
Glucose, mM	3.50^ab^	3.44^b^	3.59^a^	3.50^ab^	0.070	0.03	0.37	0.64	–
Urea, mM	3.58	3.79	3.98	3.86	0.218	0.27	0.07	0.43	–
l-Lactate, mM	1.55	1.15	1.27	1.13	0.238	0.35	0.17	0.40	–
BHB, mM	0.630	0.760	0.802	0.714	0.088	0.44	0.27	0.26	–
NEFA, µEq/L	105^a^	100^a^	120^ab^	187^b^	16.1	0.02	0.04	0.04	–
Serum biomarkers of health status^1^									
Plasma total T4, nmol/L^1^	67.2^ab^	69.4^a^	60.5^b^	59.0^b^	2.55	0.01	0.01	0.08	–
AST, U/L	44.3	40.0	45.1	30.4	4.71	0.11	0.25	0.49	< 80
g-GT, U/L	20.8	21.5	22.6	18.0	3.29	0.16	0.74	0.18	< 20
GLDH, U/L	12.5	11.2	12.8	4.4	2.75	0.09	0.22	0.30	< 8
Total bilirubin, µmol/L	0.825^b^	0.950^ab^	0.900^b^	1.37^a^	0.195	0.03	0.07	0.29	< 5.0
Total protein, g/L	69.6	70.6	72.2	67.0	1.89	0.17	0.67	0.04	60–80
Albumin, g/L	37.0	37.4	38.4	35.1	1.31	0.23	0.87	0.28	30–40
Bile acids, µmol/L	29.3	23.2	30.0	19.9	9.41	0.80	0.68	0.99	15–80

*BHB* β-OH-butyrate, *NEFA* non-esterified fatty acids, *T4* thyroxine hormone, *AST* aspartate aminotransferase, *g-GT* gamma-glutamyl transferase, and *GLDH* glutamate dehydrogenase.

^ab^Values within the same line with different superscripts differ (P < 0.05). Reference values were obtained from the commercial laboratory Laboklin Laboratory for Clinical Diagnostics GmbH & Co. KG (Bad Kissingen, Germany).

^1^Parameter measured in serum unless otherwise stated.

## Discussion

### Iodoform is a potent anti-methanogen

Iodoform is a potent CH_4_ inhibitor as increasing dose had large decreasing effects on both daily CH_4_ production, CH_4_ yield, and CH_4_ intensity. Similar results have been reported from studies investigating the use of other halomethanes as anti-methanogenic feed additives in ruminants^6,16,20^. Reductions in CH_4_ emission were accompanied by a log10 fold reduction in total methanogens and a 1.6 log10 fold reduction in the specific methanogen genera *Methanobrevibacter* and *Methanosphaera*. However, rapid changes in diurnal CH_4_ emission around dosing of iodoform could indicate that iodoform acted primarily to suppress the metabolic activity of methanogens by inhibiting a pathway of importance for their energy metabolism. Moreover, despite their primary role in methanogenesis, a meta-analysis by Newbold et al.^55^ found that the abundance of archaea only had a weak correlation with CH_4_ emissions from individual animals. As this is the first published study testing iodoform when supplemented to dairy cows, the authors do not have evidence to prove that the effects of iodoform had reached a stationary state. However, DMI was recorded during the last week of each period and feed intake throughout the period, and those parameters appeared to have stabilized. Moreover, Machado et al.^56^ found that rumen fermentation pattern and bacterial community composition on average stabilized within 7–8 days. This complies with the results from Fig. S2 supplementary as the microbial composition of the cow that received the highest iodoform dose in the first period (1080 mg/day; data excluded from the dataset) appeared to have recovered in the following period and stayed stable throughout the remaining periods, indicating that the adaptation period of 7-d was long enough.

Interestingly, while counts of *Methanobrevibacter* and *Methanosphaera*, which both are dominant members of the rumen archaeal community^57^, decreased with increasing doses of iodoform, the counts of *Methanomassiliicoccales* order, formerly called rumen cluster C (RCC), were less affected by treatment. The *Methanomassiliicoccales* only showed a tendency to decrease linearly with increasing iodoform dose measured by qPCR. However, *Ca._Methanomethylophilus* genus, a member of the *Methanomassiliicoccales* order, had a 2.7 fold decrease in the 800 mg/day iodoform treatment compared with the control in the amplicon sequences. The dramatic reduction of copies seen for *Methanobrevibacter* and *Methanosphaera* with qPCR but not in the amplicon sequences can be explained by differences in the primers used, where universal prokaryotic primers designed for amplicon sequencing may have limited coverage of archaea^58^. In a study by Knight et al.^20^, the number of *Methanomassiliicoccales* even seemed to increase, when cows were supplemented with chloroform. These changes in methanogen communities may be ascribed to several factors. Halomethanes have been suggested to inhibit the function of corrinoid enzymes and the cobamide-dependent methyl group transfer in the methanogenesis process^12,13^. Thus, they can competitively inhibit methyl-coenzyme M reductase activity^59^. While some methanogens are able to synthesize an archaea specific co-factor needed in this process, namely coenzyme M (CoM), other methanogens, such as *Methanobrevibacter*, are dependent on other methanogens to synthesize CoM. This might explain why *Methanobrevibacter* seems to be more sensitive to the use of halomethanes^60^.

The changes in abundance of the different methanogen genera might also be explained by differences in sensitivity towards allosteric inhibition of enzymatic activity by halomethanes depending on the species-specific pathways leading to CH_4_ formation. *Methanosphaera* use only methanol and H_2_ as substrates, while *Methanobrevibacter* uses the hydrogenotrophic pathway in which CO_2_, formate and H_2_ are metabolized to form CH_4_. In contrast, *Methanomassiliicoccales* perform a methyl-dependent hydrogenotrophic methanogenesis by reducing methyl-compounds with H_2_ as an electron donor^60,61^. Irrespectively of this, *Methanomassiliicoccales* typically represents around 9.5% of rumen methanogens while the *Methanobacteriales* order containing *Methanobrevibacter* and *Methanosphaera* are the numerically dominant genera^62^, which can explain why the significant reduction in the latter two genera was accompanied by the large reduction in methane.

### Hydrogenotrophic pathways in the rumen

Similar to findings in other studies investigating anti-methanogenic feed additives^7,18,20^, H_2_ emission increased dramatically in our study with increasing inhibition of the methanogenesis. However, based on stoichiometric calculations only a part of the theoretical excess of H_2_ upon suppression of CH_4_ formation was recovered as emitted H_2_. Cows supplemented 0 mg/day of iodoform yielded 1170 mmol CH_4_/kg DMI and 9.61 mmol H_2_/kg DMI, while cows fed 800 mg/day emitted 395 mmol CH_4_/kg DMI and 1209 mmol H_2_/kg DMI. Production of 1 mol CH_4_ consumes 4 mol H_2_^63^, and hence a reduction in CH_4_ yield of 776 mmol/kg DMI should have given rise to a theoretical increase in H_2_ emission of approximately 3100 mmol/kg DMI. Thus, in our study, H_2_ yield only increased by around 40% of the theoretical generated excess of H_2_ in the rumen. This implies that a substantial proportion of H_2_ must have been re-directed into other H_2_-consuming biological processes in the rumen and/or a change in microbiota towards a lower net H_2_ production have occured.

Since propionate synthesis offers an alternative pathway for utilization of excess H_2_ when the methanogenesis is inhibited^4^, we had expected to find a higher proportion of propionate in total VFA in rumen fluid at the expense of acetate. However, no effect was observed on the proportion of propionate in total VFA. The formation of valerate can also act as a H_2_-sink in the rumen^64,65^, and therefore some of the excess H_2_ might have been incorporated in valerate. Thus, the tendency to a linear increase in proportion of valerate with increasing dose of iodoform may indicate that other hydrogenotrophic pathways were recruited when iodoform was administered to the cows.

Interestingly, we also observed an increase in the abundance in the rumen of the genus *Ruminobacter* in the family *Succinivibrionaceae* when iodoform was supplemented. Increased abundances of a second genus in *Succinivibrionaceae* (Succinivibrionaceae_UCG-002) were also detected when 640 and 800 mg/day iodoform were supplemented. Members of the *Succinivibrionaceae* family have been associated with high feed efficiency in dairy cows^66^. Additionally, Danielsson et al.^67^ reported that the abundance of unclassified *Succinivibrionaceae* in the rumen was correlated with reduced CH_4_ emissions, and Mccabe et al.^68^ showed that increased abundance of methanogens were negatively correlated with the abundance of *Succinivibrionaceae* in bulls. Furthermore, Pope et al.^69^ speculated that an explanation for why wallabies produce only one fifth the amount of CH_4_ as ruminants on a fibrous plant based diet could be due to a higher abundance of *Succinivibrionaceae*. Members of *Succinivibrionaceae* family utilize H_2_ to produce succinate^70^. Thus, the increased abundance of *Succinivibrionaceae* genera, when cows were supplemented with iodoform, suggests that upregulation and growth of these bacteria also became an alternative H_2_-sink pathway.

### Rumen fermentation dynamics and digestive processes

A surplus of electron donors from H_2_ accumulation may suppress rumen fermentation and microbial synthesis, and thereby indirectly influence the DMI^4,71^. Microbial produced H_2_ has also been hypothesized to be responsible for the reducing conditions of the ruminal environment^72^. In line with this, increasing doses of iodoform were associated with a marked linear reduction in redox potential as well as increased rumen pH, which could indicate changes in ruminal microbial activity and fermentation dynamics^73^.

Iodoform did not seem to kill off the bacteria as total microbial species richness and qPCR count of bacteria were unaffected by treatment, but analysis of beta diversity showed an alteration of the composition of the microbiome with a clear separation of the 0 mg/day group from the 640 and 800 mg/day group, whereas the 320 mg/day group was placed in between. It could be speculated that the microbiota might have changed towards lower net H_2_ production.

A change in ruminal microbial population may also contribute to explain the observed depressions in DMI, and the significant or tendencies to linear decreases in ruminal digestibility of all nutrients, except NDF, with increasing dose of iodoform. Literature on the function of *Tuzzerella¸* belonging to the family *Lachnospiraceae*, is limited. However, many members of the *Lachnospiraceae* family have cellulolytic activity and are related to butyrate production^74^. Higher abundance of *Tuzzerella* on the two highest doses compared to 0 mg/day might explain why NDF was the only nutrient not showing a linear decrease in ruminal digestibility with increasing dose of iodoform. Increasing proportions of isobutyrate and isovalerate in total VFA with increasing dose of iodoform might have facilitated growth of this order as isoacids are important for the growth and activity of especially cellulolytic ruminal bacteria^75^. Higher abundance of *Tuzzerella* might also explain the increased proportion of butyrate in the rumen fluid with increasing dose of iodoform.

The ruminal digestibility of CP was negative for all treatments, but to a higher extent with increasing dose of iodoform. Increased recirculation of urea-N to the rumen with increasing dose of iodoform might explain such a change. In ruminants, urea-N can be recycled to the rumen via the saliva or through the rumen wall. This recirculation can become an important source of nitrogen for microbial growth when the amount of rumen degradable protein, and hence when concentrations of the N-substrate NH_3,_ is insufficient^76^. The concentration of NH_3_ in the rumen is determined by the relative rate of NH_3_ formation from degradation of either feed protein or urea in saliva, relative to the rate of utilization for bacterial protein synthesis, uptake across the rumen wall, and wash-out from the rumen^77^. In our study, we observed a linear decrease in concentration of NH_3_ with increasing dose of iodoform. This could indicate a more efficient microbial protein synthesis as more NH_3_ is converted into microbial protein, and hence explain the observed effects on ruminal CP digestibility with increasing dose of iodoform. Conversely, studies found a minimum required level of NH_3_ for rumen microbes to grow well ranging from 4.7–5.0 mg/100 mL with on optimal level ranging between 8.5 and 30.0 mg/100 mL^78,79^. Concentrations of NH_3_ in rumen fluid from cows fed 0 and 800 mg/day iodoform was 5.5 and 2.76 mM, corresponding to 9.37 and 4.70 mg/100 mL, respectively. Thus, the ruminal level of NH_3_ on the highest dose of iodoform was only around a minimum level. Consequently, the lower NH_3_ concentration with increasing iodoform could, on the other hand, also indicate a disturbed ruminal microbiota.

Reduced feed intake would usually be expected to result in a lower passage rate of feed out of the rumen, which leads to an increased retention time and thereby more efficient microbial digestion^80,81^. Therefore, the lower DMI with increasing dose of iodoform might have had an increasing effect on ruminal digestibility and also contribute to explain why ruminal NDF digestibility was unaffected by treatments.

In the small intestine, increasing dose of iodoform was accompanied by higher digestibility of all nutrients, except starch, which counteracted the changes in ruminal digestibility. Therefore, total tract digestibility of all nutrients were unaffected by treatment. Similar results were reported by Knight et al.^20^ and Mitsumori et al.^16^, who investigated chloroform and bromochloromethane as CH_4_ mitigating feed additives in cows and goats, respectively.

### Milk synthesis and metabolic and health status of cows

Several parameters indicated that energy and protein balance of cows became increasingly negative with increasing dose of iodoform due to the reduced DMI. The observed linear increases in fat percentage in milk with increasing doses of iodoform combined with a higher proportion of long-chained fatty acids in milk are indicative of increased mobilization of fat from adipose tissue^82^. The observed increased levels of NEFA in the serum also indicate increased mobilization of fat^83^. Milk fatty acids with 18 or more carbons are not synthesized de novo within the mammary gland, and in the present study they most likely derived from mobilization of fatty acids from adipose tissue as dietary intake of lipids did not increase^84^. Furthermore, the proportion of fat or protein in oxidative pathways was increased in cows upon iodoform administration as indicated by a drop in RQ below 1 at the highest dose of iodoform^85^.

Apart from the increased mammary uptake of long-chain FA, a reduced mammary de novo synthesis of FA may also have contributed to the observed changes in milk FA profiles. Acetate and to lesser extent BHB are the main precursors for de novo FA synthesis, which in the mammary gland results in production of short- and medium-chain FA with 4–16 carbon atoms^84^. Lower ruminal concentrations and hence absorption of acetate are the likely explanations for the observed reduced proportion of MCFA in milk fat with increasing dose of iodoform.

In addition to a more negative energy balance, there were also indications that the protein balance of cows became more negative, and at the highest doses of iodoform potentially leading to a net-mobilization of amino acids from body proteins. To compensate for a low nitrogen intake, amino acids released from peripheral tissues can, in the liver, undergo amidation or transamination resulting in incorporation of N into urea^86^. The tendency for a linear increase in urea levels in serum could indicate that this occurred to an increasing extent with increasing doses of iodoform. This would be consistent with an increased recirculation of urea to the rumen via saliva, which the more negative ruminal digestibility of CP with increasing dose of iodoform could imply, as previously discussed.

Breakdown of skeletal muscle tissue results in release of creatinine, which is subsequently excreted into urine^87^. Løvendahl and Sehested^88^ reported creatinine concentrations in urine of healthy cows in positive energy balance similar to the levels observed in the present study for the two lowest doses while the levels for the two highest doses of iodoform exceeded the expected urine concentrations of cows in positive energy balances. Urine creatinine level can also be affected by water intake^89^, but percentage of total water intake excreted in urine was unaffected by treatments in our study.

The daily levels of supplemented iodine exceeded the maximum total limit (50 mg I/day) set by the U.S. Food and Drug Administration regulations. Iodine is a required nutrient in the synthesis of thyroid hormones, and this synthesis can be used as an indicator of thyroid function^90^. High iodine levels can down-regulate synthesis of thyroxine or T4 due to negative feedback mechanisms in the hypothalamic-pituitary-thyroid hormone axis^91^. Assuming that iodoform can be metabolized in both the rumen and liver resulting in release of ionized iodine, increased intake of iodine might explain linear reductions in plasma T4 with increasing dose of iodoform. However, the levels remained within a normal range (54–110 nmol/L) for healthy cattle^92^. Therefore, thyroid function did not seem to be impaired by iodoform at any dose given for the duration it was supplemented to the cows in the experiment. Plasma levels of T4 in cattle are also influenced by a variety of other factors, including feed intake^93^. Thus, the marked depression in DMI may also have contributed to explain decreases in T4 concentration with increasing doses of iodoform.

Numerous halomethane analogs have been found to cause liver injuries in terms of damaged or necrotic hepatocytes in different animal species^94–96^. An evaluation of liver function should be based on an assessment of several hepatic enzymes and indicators of hepatic injury or disease^97^. Gamma-glutamyl transferase (g-GT) is commonly used in combination with other hepatic enzymes as indexes for liver dysfunction^97^. No treatment effect was found on the level of g-GT, but all treatment groups, except 800 mg/day, had g-GT levels slightly above the normal reference range indicated by the commercial laboratory. Similarly, the level of GLDH also exceeded the reference range from the commercial laboratory for all treatment groups, except 800 mg/day. Nevertheless, the levels of both enzymes for all treatment groups were within the range reported for clinically healthy Holstein cows in other studies^98,99^. In agreement with these findings in the present study, Lanigan et al.^100^ did not observe any significant signs of liver dysfunction, when similar amounts of iodoform per kg live weight were supplemented to sheep. However, it should be noted that the present study only investigated short-term use of iodoform.

## Conclusions

Iodoform had a dramatic and dose-dependent suppressing effect on daily CH_4_ emission, yield, and intensity from dairy cows, most likely caused by a depression of metabolic activity of the methanogens. The theoretical excess of H_2_ resulting from the reduction of CH_4_ synthesis was only partially recovered as emitted H_2_ since alternative hydrogen-sink pathways were activated through upregulation of hydrogenotrophic bacteria and/or a change in microbiota towards a lower net H_2_ production. For all nutrients, except NDF, rumen digestibility was depressed, and the marked depressions in DMI might be ascribed to accumulation of H_2_ in rumen headspace or undesirable effects of iodoform on microbial fermentation, although total number of ruminal bacteria was unaffected by treatment. Due to a substantial shift from ruminal to small intestinal digestibility, overall digestibility for all nutrients was unaffected by iodoform. The supplemented doses of iodoform did not appear to have negative impacts on thyroid or liver function of the cows for the duration of the experiment, but there were several indications of a more negative energy and protein balance with increasing dose of iodoform, which contributed to explain why milk production was less negatively affected by iodoform than DMI.

### Supplementary Information

Supplementary Information.

## Data Availability

The data were exhibited in the main manuscript and supplemental materials. Raw microbiome sequence reads are deposited in the NCBI short-read archive database under BioProject ID: PRJNA906944 (https://www.ncbi.nlm.nih.gov/bioproject/PRJNA906944).

## Abbreviations

ADF: Acid detergent fiber
ADL: Acid detergent lignin
AST: Aspartate aminotransferase
BHB: β-OH-butyrate
C: Carbon
CO_2_: Carbon dioxide
CoM: Coenzyme M
CP: Crude protein
DMI: Dry matter intake
FA: Fatty acids
g-GT: Gamma-glutamyl transferase
GLDH: Glutamate dehydrogenase
H_2_: Hydrogen
LCFA: Long-chain fatty acids
MCFA: Medium-chain fatty acids
CH_4_: Methane
NDF: Neutral detergent fiber
NEFA: Non-esterified fatty acids
PMR: Partial mixed ration
RQ: Respiratory coefficient
T4: Thyroxine
VFA: Volatile fatty acids

## References

[1] Gerber PJ, Steinfeld H, Henderson B, Mottet A, Opio C, Dijkman J (2013). Talking Climate Change Through Livestock—A Global Assesment of Emissions and Mitigation Opportunities.

[2] Myhre G, Shindell D, Bréon FM, Collins W, Fuglestvedt J, Huang J, Stocker TF, Qin D, Plattner G-K, Tignor M, Allen SK, Boschung J, Nauels A, Xia Y, Bex V, Midgley PM (2013). Anthropogenic and natural radiative forcing. Climate Change 2013: The Physical Science Basis Contribution of Working Group I to the Fifth Assessment Report of the Intergovernmental Panel on Climate Change.

[3] Ellis JL, Dijkstra J, Kebreab E, Bannink A, Odongo NE, McBride BW (2008). Aspects of rumen microbiology central to mechanistic modelling of methane production in cattle. J. Agric. Sci..

[4] McAllister TA, Newbold CJ (2008). Redirecting rumen fermentation to reduce methanogenesis. Aust. J. Exp. Agric..

[5] Janssen PH (2010). Influence of hydrogen on rumen methane formation and fermentation balances through microbial growth kinetics and fermentation thermodynamics. Anim. Feed Sci. Technol..

[6] Martinez-Fernandez G, Denman SE, Yang C, Cheung J, Mitsumori M, McSweeney CS (2016). Methane inhibition alters the microbial community, hydrogen flow, and fermentation response in the rumen of cattle. Front. Microbiol..

[7] Melgar A, Welter KC, Nedelkov K, Martins CMMR, Harper MT, Oh J (2020). Dose-response effect of 3-nitrooxypropanol on enteric methane emissions in dairy cows. J. Dairy Sci..

[8] Lanigan G (1972). Metabolism of pyrrolizidine alkaloids in the ovine rumen. IV. Effects of chloral hydrate and halogenated methanes on rumen methanogenesis and alkaloid metabolism in fistulated sheep. Aust. J. Agric. Res..

[9] Czerkawski JW, Breckenridge G (1975). New inhibitors of methane production by rumen micro-organisms. Development and testing of inhibitors in vitro. Br. J. Nutr..

[10] Czerkawski JW, Breckenridge G (1975). New inhibitors of methane production by rumen micro-organisms. Experiments with animals and other practical possibilities. Br. J. Nutr..

[11] Chalupa W, Ruckebusch Y, Thivend P (1980). Chemical control of rumen microbial metabolism. Digestive Physiology and Metabolism in Ruminants: Proceedings of the 5th International Symposium on Ruminant Physiology, Held at Clermont—Ferrand, on 3rd–7th September, 1979.

[12] Glasson CRK, Kinley RD, de Nys R, King N, Adams SL, Packer MA (2022). Benefits and risks of including the bromoform containing seaweed *Asparagopsis* in feed for the reduction of methane production from ruminants. Algal Res..

[13] Wood JM, Kennedy FS, Wolfe RS (1968). Reaction of multihalogenated hydrocarbons with free and bound reduced vitamin B12. Biochemistry.

[14] Patra A, Park T, Kim M, Yu Z (2017). Rumen methanogens and mitigation of methane emission by anti-methanogenic compounds and substances. J. Anim. Sci. Biotechnol..

[15] Yu Z, Smith GB (2000). Inhibition of methanogenesis by C1- and C2-polychlorinated aliphatic hydrocarbons. Environ. Toxicol. Chem..

[16] Mitsumori M, Shinkai T, Takenaka A, Enishi O, Higuchi K, Kobayashi Y (2012). Responses in digestion, rumen fermentation and microbial populations to inhibition of methane formation by a halogenated methane analogue. Br. J. Nutr..

[17] Goel G, Makkar HPS, Becker K (2009). Inhibition of methanogens by bromochloromethane: Effects on microbial communities and rumen fermentation using batch and continuous fermentations. Br. J. Nutr..

[18] Tomkins NW, Colegate SM, Hunter RA (2009). A bromochloromethane formulation reduces enteric methanogenesis in cattle fed grain-based diets. Anim. Prod. Sci..

[19] ECHA. *Information on Chemicals*. https://echa.europa.eu/information-on-chemicals. (Accessed 28 October 2022) (European Chemicals Agency, 2022).

[20] Knight T, Ronimus RS, Dey D, Tootill C, Naylor G, Evans P (2011). Chloroform decreases rumen methanogenesis and methanogen populations without altering rumen function in cattle. Anim. Feed Sci. Technol..

[21] Martinez-Fernandez G, Duval S, Kindermann M, Schirra HJ, Denman SE, McSweeney CS (2018). 3-NOP vs halogenated compound: Methane production, ruminal fermentation and microbial community response in forage fed cattle. Front. Microbiol..

[22] Fang X, Park S, Saito T, Tunnicliffe R, Ganesan AL, Rigby M (2019). Rapid increase in ozone-depleting chloroform emissions from China. Nat. Geosci..

[23] Golden RJ, Holm SE, Robinson DE, Julkunen PH, Reese EA (1997). Chloroform mode of action: Implications for cancer risk assessment. Regul. Toxicol. Pharmacol..

[24] Volden H (2011). NorFor, the Nordic Feed Evaluation System.

[25] Hellwing ALF, Lund P, Weisbjerg MR, Brask M, Hvelplund T (2012). Technical note: Test of a low-cost and animal-friendly system for measuring methane emissions from dairy cows. J. Dairy Sci..

[26] ThermoFisher. *DNA Copy Number Calculator* (Accessed 24 January 2022) (2022).

[27] Goberna M, Gadermaier M, García C, Wett B, Insam H (2010). Adaptation of methanogenic communities to the cofermentation of cattle excreta and olive mill wastes at 37 degrees C and 55 degrees C. Appl. Environ. Microbiol..

[28] Mihajlovski A, Doré J, Levenez F, Alric M, Brugère JF (2010). Molecular evaluation of the human gut methanogenic archaeal microbiota reveals an age-associated increase of the diversity. Environ. Microbiol. Rep..

[29] Poulsen M, Schwab C, Jensen BB, Engberg RM, Spang A, Canibe N (2013). Methylotrophic methanogenic *Thermoplasmata* implicated in reduced methane emissions from bovine rumen. Nat. Commun..

[30] Lee DH, Zo YG, Kim SJ (1996). Nonradioactive method to study genetic profiles of natural bacterial communities by PCR-single-strand-conformation polymorphism. Appl. Environ. Microbiol..

[31] Noel SJ, Olijhoek DW, McLean F, Løvendahl P, Lund P, Højberg O (2019). Rumen and fecal microbial community structure of Holstein and Jersey dairy cows as affected by breed, diet, and residual feed intake. Animals (Basel).

[32] Klindworth A, Pruesse E, Schweer T, Peplies J, Quast C, Horn M (2013). Evaluation of general 16S ribosomal RNA gene PCR primers for classical and next-generation sequencing-based diversity studies. Nucleic Acids Res..

[33] Bolyen E, Rideout JR, Dillon MR, Bokulich NA, Abnet CC, Al-Ghalith GA (2019). Reproducible, interactive, scalable and extensible microbiome data science using QIIME 2. Nat. Biotechnol..

[34] Callahan BJ, McMurdie PJ, Rosen MJ, Han AW, Johnson AJA, Holmes SP (2016). DADA2: High-resolution sample inference from Illumina amplicon data. Nat. Methods.

[35] Katoh K, Misawa K, Kuma K, Miyata T (2002). MAFFT: A novel method for rapid multiple sequence alignment based on fast Fourier transform. Nucleic Acids Res..

[36] Price MN, Dehal PS, Arkin AP (2010). FastTree 2-approximately maximum-likelihood trees for large alignments. PLoS ONE.

[37] AOAC International (2000). Official Methods of Analysis.

[38] Stoldt W (1952). Vorschlag zur Vereinheitlichung der Fettbestimmung in Lebensmitteln. Fette und Seifen.

[39] ANKOM Technology. *Analytical Methods*. https://www.ankom.com/analytical-methods-support/fiber-analyzer-a2000 (Accessed 25 October 2022) (2017).

[40] Mertens DR (2002). Gravimetric determination of amylase-treated neutral detergent fiber in feeds with refluxing in beakers or crucibles: Collaborative study. J. AOAC Int..

[41] Kristensen NB, Storm A, Raun BML, Røjen BA, Harmon DL (2007). Metabolism of silage alcohols in lactating dairy cows. J. Dairy Sci..

[42] Myers WD, Ludden PA, Nayigihugu V, Hess BW (2004). Technical note: A procedure for the preparation and quantitative analysis of samples for titanium dioxide. J. Anim. Sci..

[43] Schürch AF, Lloyd LE, Crampton EW (1950). The use of chromic oxide as an index for determining the digestibility of a diet: Two figures. J. Nutr..

[44] Mason M (1983). Determination of glucose, sucrose, lactose, and ethanol in foods and beverages, using immobilized enzyme electrodes. J. Assoc. Off. Anal. Chem..

[45] Schwarz D, Bak MR, Hansen PW (2022). Development of global fatty acid models and possible applications. Int. J. Dairy Technol..

[46] Harano Y, Ohtsuki M, Ida M, Kojima H, Harada M, Okanishi T (1985). Direct automated assay method for serum or urine levels of ketone bodies. Clin. Chim. Acta.

[47] Sjaunja LO, Baevre L, Junkkarinen L, Pedersen J, Setala J (1991). A Nordic Proposal for an Energy Corrected Milk (ECM) Formula.

[48] Bates D, Mächler M, Bolker B, Walker S (2015). Fitting linear mixed-effects models using lme4. J. Stat. Softw..

[49] McMurdie PJ, Holmes S (2013). Phyloseq: An R package for reproducible interactive analysis and graphics of microbiome census data. PLoS ONE.

[50] Andersen KS, Kirkegaard RH, Karst SM, Albertsen M (2018). Ampvis2: An R package to analyse and visualise 16S rRNA amplicon data. BioRxiv.

[51] Oksanen, J. *et al*. *Vegan: Community Ecology Package. Software*. http://CRANR-project.org/package=vegan (2012).

[52] Wickham, H., Navarro, D. & Pedersen, T. L. *Elegant Graphics for Data Analysis: ggplot2. Software*. https://ggplot2tidyverse.org (2008).

[53] Love MI, Huber W, Anders S (2014). Moderated estimation of fold change and dispersion for RNA-seq data with DESeq2. Genome Biol..

[54] Benjamini Y, Hochberg Y (1995). Controlling the false discovery rate: A practical and powerful approach to multiple testing. J. R. Stat. Soc. Ser. B Stat. Methodol..

[55] Newbold CJ, de la Fuente G, Belanche A, Ramos-Morales E, McEwan NR (2015). The role of ciliate protozoa in the rumen. Front. Microbiol..

[56] Machado MG, Detmann E, Mantovani HC, Valadares SC, Bento CBP, Marcondes MI (2016). Evaluation of the length of adaptation period for changeover and crossover nutritional experiments with cattle fed tropical forage-based diets. Anim. Feed Sci. Tech..

[57] Janssen PH, Kirs M (2008). Structure of the archaeal community of the rumen. Appl. Environ. Microbiol..

[58] Bahram M, Anslan S, Hildebrand F, Bork P, Tedersoo L (2019). Newly designed 16S rRNA metabarcoding primers amplify diverse and novel archaeal taxa from the environment. Environ. Microbiol. Rep..

[59] Nollet L, Demeyer D, Verstraete W (1997). Effect of 2-bromoethanesulfonic acid and *Peptostreptococcus productus* ATCC 35244 addition on stimulation of reductive acetogenesis in the ruminal ecosystem by selective inhibition of methanogenesis. Appl. Environ. Microbiol..

[60] Pitta D, Indugu N, Narayan K, Hennessy M (2022). Symposium review: Understanding the role of the rumen microbiome in enteric methane mitigation and productivity in dairy cows. J. Dairy Sci..

[61] Thauer RK, Kaster A-K, Seedorf H, Buckel W, Hedderich R (2008). Methanogenic archaea: Ecologically relevant differences in energy conservation. Nat. Rev. Microbiol..

[62] Henderson G, Cox F, Ganesh S, Jonker A, Young W, Janssen PH (2015). Rumen microbial community composition varies with diet and host, but a core microbiome is found across a wide geographical range. Sci. Rep..

[63] Boadi D, Benchaar C, Chiquette J, Massé D (2004). Mitigation strategies to reduce enteric methane emissions from dairy cows: Update review. Can. J. Anim. Sci..

[64] Ungerfeld EM (2015). Shifts in metabolic hydrogen sinks in the methanogenesis-inhibited ruminal fermentation: A meta-analysis. Front. Microbiol..

[65] Guyader J, Ungerfeld EM, Beauchemin KA (2017). Redirection of metabolic hydrogen by inhibiting methanogenesis in the rumen simulation technique (RUSITEC). Front. Microbiol..

[66] Indugu N, Vecchiarelli B, Baker LD, Ferguson JD, Vanamala JKP, Pitta DW (2017). Comparison of rumen bacterial communities in dairy herds of different production. BMC Microbiol..

[67] Danielsson R, Dicksved J, Sun L, Gonda H, Müller B, Schnürer A (2017). Methane production in dairy cows correlates with rumen methanogenic and bacterial community structure. Front. Microbiol..

[68] McCabe MS, Cormican P, Keogh K, O’Connor A, O’Hara E, Palladino RA (2015). Illumina MiSeq phylogenetic amplicon sequencing shows a large reduction of an uncharacterised *Succinivibrionaceae* and an increase of the *Methanobrevibacter gottschalkii* clade in feed restricted cattle. PLoS ONE.

[69] Pope PB, Smith W, Denman SE, Tringe SG, Barry K, Hugenholtz P (2011). Isolation of *Succinivibrionaceae* implicated in low methane emissions from Tammar wallabies. Science.

[70] Difford GF, Plichta DR, Løvendahl P, Lassen J, Noel SJ, Højberg O (2018). Host genetics and the rumen microbiome jointly associate with methane emissions in dairy cows. PLoS Genet..

[71] van Lingen HJ, Plugge CM, Fadel JG, Kebreab E, Bannink A, Dijkstra J (2016). Thermodynamic driving force of hydrogen on rumen microbial metabolism: A theoretical investigation. PLoS ONE.

[72] Barry TN, Thompson A, Armstrong DG (1977). Rumen fermentation studies on two contrasting diets. 1. Some characteristics of the in vivo fermentation, with special reference to the composition of the gas phase, oxidation/reduction state and volatile fatty acid proportions. J. Agric. Sci..

[73] Huang Y, Marden PJ, Benchaar C, Julien C, Auclair E, Bayourthe C (2017). Quantitative analysis of the relationship between ruminal redox potential and pH in dairy cattle: Influence of dietary characteristics. Agric. Sci..

[74] Dušková D, Marounek M (2001). Fermentation of pectin and glucose, and activity of pectin-degrading enzymes in the rumen bacterium *Lachnospira multiparus*. Appl. Microbiol..

[75] Lee C, Copelin JE, Park T, Mitchell KE, Firkins JL, Socha MT (2021). Effects of diet fermentability and supplementation of 2-hydroxy-4-(methylthio)-butanoic acid and isoacids on milk fat depression: 2. Ruminal fermentation, fatty acid, and bacterial community structure. J. Dairy Sci..

[76] Mutsvangwa T, Davies KL, McKinnon JJ, Christensen DA (2016). Effects of dietary crude protein and rumen-degradable protein concentrations on urea recycling, nitrogen balance, omasal nutrient flow, and milk production in dairy cows. J. Dairy Sci..

[77] Apajalahti J, Vienola K, Raatikainen K, Holder V, Moran CA (2019). Conversion of branched-chain amino acids to corresponding isoacids—An in vitro tool for estimating ruminal protein degradability. Front. Vet. Sci..

[78] Satter LD, Slyter LL (1974). Effect of ammonia concentration of rumen microbial protein production in vitro. Br. J. Nutr..

[79] McDonald P, Edwards RA, Greenhalgh JFD, Morgan CA, Sinclair LA, Wilkinson RG (2012). Animal Nutrition.

[80] Tyrrell HF, Moe PW (1975). Effect of intake on digestive efficiency. J. Dairy Sci..

[81] Potts SB, Boerman JP, Lock AL, Allen MS, VandeHaar MJ (2017). Relationship between residual feed intake and digestibility for lactating Holstein cows fed high and low starch diets. J. Dairy Sci..

[82] Börner S, Derno M, Hacke S, Kautzsch U, Schäff C, Than S (2013). Plasma ghrelin is positively associated with body fat, liver fat and milk fat content but not with feed intake of dairy cows after parturition. J. Endocrinol..

[83] Contreras GA, O’Boyle NJ, Herdt TH, Sordillo LM (2010). Lipomobilization in periparturient dairy cows influences the composition of plasma nonesterified fatty acids and leukocyte phospholipid fatty acids. J. Dairy Sci..

[84] Jensen RG (2002). The composition of bovine milk lipids: January 1995 to December 2000. J. Dairy Sci..

[85] Kim DH, McLeod KR, Klotz JL, Koontz AF, Foote AP, Harmon DL (2013). Evaluation of a rapid determination of fasting heat production and respiratory quotient in Holstein steers using the washed rumen technique. J. Anim. Sci..

[86] Sarraseca A, Milne E, Metcalf MJ, Lobley GE (1998). Urea recycling in sheep: Effects of intake. Br. J. Nutr..

[87] Megahed AA, Hiew MWH, Ragland D, Constable PD (2019). Changes in skeletal muscle thickness and echogenicity and plasma creatinine concentration as indicators of protein and intramuscular fat mobilization in periparturient dairy cows. J. Dairy Sci..

[88] Løvendahl P, Sehested J (2016). Short communication: Individual cow variation in urinary excretion of phosphorus. J. Dairy Sci..

[89] Franz S, Skopp G, Boettcher M, Musshoff F (2019). Creatinine excretion in consecutive urine samples after controlled ingestion of water. Drug Test Anal..

[90] NRC (2021). Nutrient Requirements of Dairy Cattle: Eighth Revised Edition.

[91] Chung HR (2014). Iodine and thyroid function. Ann. Pediatr. Endocrinol. Metab..

[92] Jackson PGG, Cockcroft PD (2002). Appendix 3—Laboratory Reference Values: Biochemistry. Clinical Examination of Farm Animals.

[93] Akasha MA, Anderson RR, Ellersieck M, Nixon DA (1987). Concentration of thyroid hormones and prolactin in dairy cattle serum and milk at three stages of lactation. J. Dairy Sci..

[94] Klingensmith JS, Mehendale HM (1981). Potentiation of brominated halomethane hepatotoxicity by chlordecone in the male rat. Toxicol. Appl. Pharmacol..

[95] Roberts SM, Harbison RD, Seng JE, James RC (1991). Potentiation of carbon tetrachloride hepatotoxicity by phenylpropanolamine. Toxicol. Appl. Pharmacol..

[96] Barakat SEDM, Ford EJH (1988). Further studies on the diagnostic value of γ-glutamyl transpeptidase and 5′-nucleotidase in cattle, sheep and horses. Res. Vet. Sci..

[97] Whitfield JB (2001). Gamma glutamyl transferase. Crit. Rev. Clin. Lab. Sci..

[98] Cozzi G, Ravarotto L, Gottardo F, Stefani AL, Contiero B, Moro L (2011). Short communication: Reference values for blood parameters in Holstein dairy cows: Effects of parity, stage of lactation, and season of production. J. Dairy Sci..

[99] Imhasly S, Naegeli H, Baumann S, von Bergen M, Luch A, Jungnickel H (2014). Metabolomic biomarkers correlating with hepatic lipidosis in dairy cows. BMC Vet. Res..

[100] Lanigan G, Payne A, Peterson J (1978). Antimethanogenic drugs and *Heliotropium europaeum* poisoning in penned sheep. Aust. J. Agric. Res..

