# Supplementary material for: Enteric methane emission of dairy cows supplemented with iodoform in a dose–response study
Source: Sci Rep. 2023 Aug 7;13:12797. doi: 10.1038/s41598-023-38149-y (PMC10406889; doi:10.1038/s41598-023-38149-y)
Supplement: Supplementary file 1 — Supplementary Information. [file 41598_2023_38149_MOESM1_ESM.docx]

# Supplementary tables

**Table S1**: Development in dry matter intake, milk yield, and gas exchange of the dairy cow supplemented with 1080 mg/d of iodoform. The treatment was stopped in the morning before feeding on day 12.

| **Day** | **11** | **12** | **13** | **14** |
| --- | --- | --- | --- | --- |
| **Daily amount of iodoform (mg/d)** | 1080 | 0 | 0 | 0 |
| **Production data** |  |  |  |  |
| Dry matter intake (kg/d) | 8.61 | 11.1 | 17.6 | 18.1 |
| ECM (kg/d) | 12.6 | 15.5 | 11.3 | 12.9 |
| Fat (%) | 5.99 | 4.77 | 5.00 | 3.72 |
| Urea (%) | 5.87 | 4.81 | 4.83 | 4.13 |
| Protein (%) | 3.32 | 3.31 | 3.43 | 3.52 |
| Lactose (%) | 5.05 | 5.12 | 5.06 | 5.13 |
| **Gas exchange** |  |  |  |  |
| CH_4_ (g/d) | 10.5 | 18.6 | 109 | 234 |
| CO_2_ (g/d) | 8597 | 8553 | 9773 | 11874 |
| O_2_ (g/d) | 7475 | 7297 | 6541 | 7808 |
| H_2_ (g/d) | 28.2 | 24.7 | 17.7 | 3.24 |
| Respiration coefficient (L/L) | 0.83 | 0.84 | 1.08 | 1.11 |

^1^Calculated ratio between carbon dioxide produced and oxygen consumed.

|  | **Iodoform supplementation** | | | |  |  |  | **P-values** | | |
| --- | --- | --- | --- | --- | --- | --- | --- | --- | --- | --- |
|  | 0 mg/d | 320 mg/d | 640 mg/d | 800 mg/d |  | SEM |  | Treatment | Linear effect | Quadratic effect |
| Total water intake, kg^1^ | 102.0 | 91.0 | 67.7 | 54.5 |  | 12.2 |  | 0.08 | <0.01 | 0.47 |
| Recorded water intake, kg | 74.6 | 67.2 | 49.2 | 39.6 |  | 9.12 |  | 0.08 | <0.01 | 0.43 |
| % of total water intake | 73.3 | 73.4 | 73.6 | 73.9 |  | 0.976 |  | 0.94 | 0.56 | 0.85 |
| Water intake from feed, kg | 27.4 | 23.8 | 18.5 | 14.9 |  | 3.15 |  | 0.07 | <0.01 | 0.59 |
| % of total water intake | 26.7 | 26.6 | 26.4 | 26.1 |  | 0.977 |  | 0.94 | 0.56 | 0.85 |
|  |  |  |  |  |  |  |  |  |  |  |
| **Outputs of water, kg** |  |  |  |  |  |  |  |  |  |  |
| Estimated water in milk, kg^2^ | 27.7^a^ | 26.9^ab^ | 22.9^ab^ | 17.6^b^ |  | 2.95 |  | 0.04 | 0.01 | 0.12 |
| % of total water intake | 27.0 | 30.0 | 34.8 | 36.1 |  | 3.48 |  | 0.07 | <0.01 | 0.87 |
| Water in faeces, kg^3^ | 43.7^a^ | 42.0^ab^ | 33.0^ab^ | 23.6^b^ |  | 4.46 |  | 0.04 | <0.01 | 0.14 |
| % of total water intake | 42.7 | 47.4 | 51.3 | 41.2 |  | 5.51 |  | 0.48 | 0.69 | 0.27 |
| Estimated water in urine, kg^4^ | 30.6 | 22.2 | 11.8 | 12.6 |  | 8.13 |  | 0.26 | 0.04 | 0.80 |
| % of total water intake | 30.3 | 22.6 | 13.9 | 22.7 |  | 7.65 |  | 0.39 | 0.18 | 0.43 |

**Table S2**: Water balance of dairy cows supplemented with four different levels of iodoform (0, 320, 640, and 800 mg/d) intra-ruminally twice daily.

^a–b^Values within the same line with different superscripts differ (P<0.05).

^1^Calculated as the sum of recorded water intake and water from feed.

^2^Calculared as kg milk subtracted content of fat, lactose, and protein, assuming a similar content of minerals.

^3^Calculared as difference between total fecal flow and DM flow.

^4^Calculated as total intake subtracted by water excreted in feces and estimated excretion in milk, ignoring an assumed similar evaporation.

**Table S3:** *Differential abundant genera identified comparing iodoform treatment group 320 with 0* ***(A)****; 640 with 0* ***(B)****, and 800 with 0* ***(C)****. Differential abundance analysis was conducted for prevalence filtered and pruned ASV counts present in at least 10 % of the samples and collapsed to genus level. Only significant differentially abundant genera are reported. Genera with an adjusted P-value (padj) ≤ 0.05, an FDR cut-off of 5% and a log2 fold change (log2FC) ≤ -2 or ≥ 2 were considered significant.*

# Supplementary figures

**
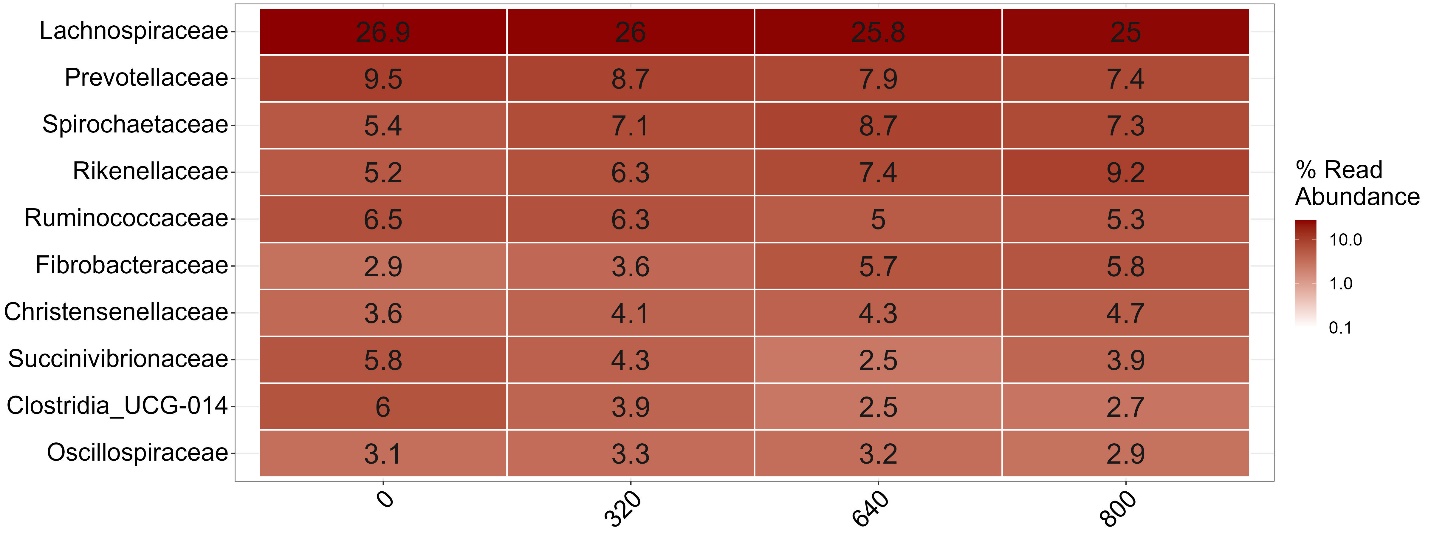
**

**Figure S1:** *Heat map of the top 10 most abundant families for each treatment across all samples. Data used were prevalence filtered, pruned, and rarefied.*


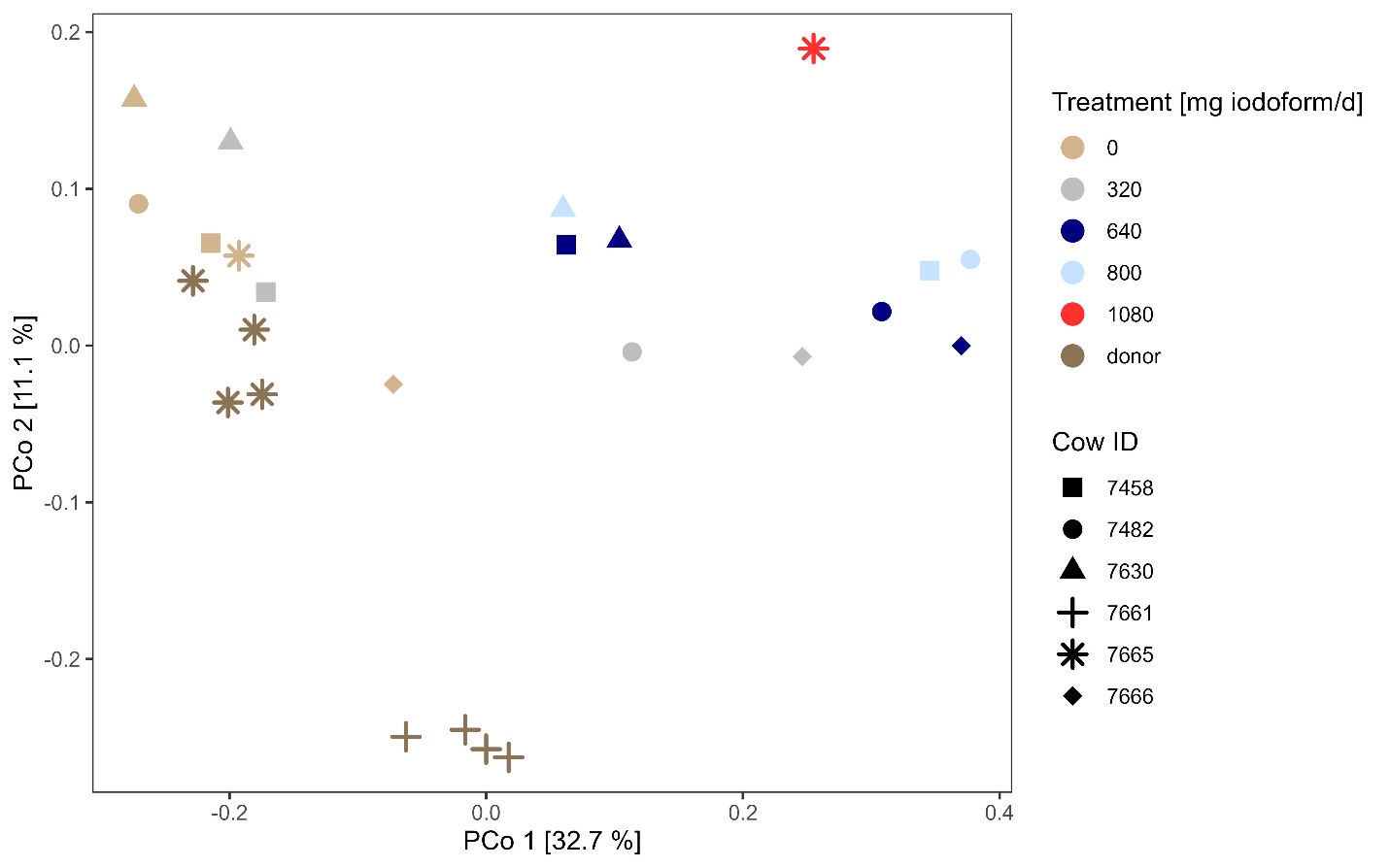


**Figure S2:**  *Principal coordinate (PCo) ordination plot based on Bray-Curtis distances representing iodoform treatment effect on rumen microbial community composition on PCo 1 and PCo 2. Bray-Curtis distances were estimated for prevalence filtered, pruned and rarefied ASV counts and colored based on iodoform treatment group: 0 mg iodoform/day, 320 mg  iodoform/day, 640 mg iodoform/day, 800 mg iodoform/day, 1080 mg iodoform/day. Cow IDs are indicated by shape. Total variance explained by each PCo is stated in parenthesis in the axis’s labels.*

*In addition to experimental cows, donor cows that were used to supply rumen fluid and content to the treatment cows have been included (indicated by star and plus-shaped data points). Donor cows were sampled twice each in the 3^rd^ and 4^th^ periods. Cow 7665 (represented by the star shape) was initially treated with 1080 mg iodoform/day in week 1 and then removed from the trial receiving no additive supplementation (0 mg iodoform/day) in week 2, after which cow 7665 was used as a donor cow (0 mg iodoform/day) and was sampled twice on each of week 3 and 4.*

*The microbiota composition of cow 7665 one week after the highest dose (period 2) was comparable to periods 3 and 4, indicating that the adaptation period, although it was short, was appropriate.*
